# Technical note: A flexible framework for precision reduction of WRF inputs and outputs to balance storage efficiency and scientific fidelity

**DOI:** 10.5194/acp-26-7261-2026

**Published:** 2026-05-27

**Authors:** Shang Wu, David C. Wong, Jiandong Wang, Yuzhi Jin, Junjun Li, Chunsong Lu

**Affiliations:** 1 State Key Laboratory of Climate System Prediction and Risk Management, China Meteorological Administration Aerosol-Cloud and Precipitation Key Laboratory, and Collaborative Innovation Center on Forecast and Evaluation of Meteorological Disasters (CIC-FEMD), Nanjing University of Information Science and Technology, Nanjing 210044, China; 2 Applied Science & Environmental Methods Division, Office of Applied Science and Environmental Solutions, US Environmental Protection Agency, Research Triangle Park, NC 27711, USA; 3 National Institute of Education (NIE), Nanyang Technological University (NTU), 637616, Singapore

## Abstract

The rapid growth of data volumes from high-resolution regional climate simulations necessitates effective storage reduction strategies that do not compromise scientific integrity. Applying lossy precision reduction prior to lossless compression provides a promising approach. However, the distinct scientific implications of reducing precision in time-varying input forcings versus prognostic model outputs remain insufficiently quantified. Using a one-year Weather Research and Forecasting (WRF) simulation, we systematically evaluate the storage benefits and downstream diagnostic impacts of retaining 5, 4, and 3 significant digits for both inputs and outputs. From a storage perspective, combining precision reduction (retaining 5–3 digits) with bzip2 compression reduces model outputs to 19.2 %–7.5 % of their original uncompressed sizes and model inputs to 52.4 %–18.5 %. Scientifically, precision-reduced inputs interact with nonlinear model dynamics and can induce spatial phase shifts in simulated meteorological systems. Although this process reduces deterministic grid-scale correspondence, the overall spatial morphology of the atmospheric structures remains largely preserved. Consequently, aggregate statistical distributions are weakly affected, especially during dynamically less active periods, rendering input precision reduction suitable for large-scale spatial aggregates and event-averaged statistical analyses. In contrast, output precision reduction acts as a static numerical filter whose impacts depend strongly on the intrinsic characteristics of individual variables. For example, regarding surface wind speed, retaining 3 significant digits still preserves an adequate error buffer. Cumulative variables, such as precipitation, progressively amplify quantization errors during temporal differencing and therefore require 4–5 significant digits to avoid artificial increments. Ultimately, this study constructs a flexible framework for WRF data compression. By dynamically tailoring precision to specific variable typologies and downstream scientific demands, the modeling community can substantially improve storage efficiency while rigorously safeguarding physical integrity.

## 1 Introduction

Climate variability and change, driven by both natural processes and human activities, have profound impacts on human societies and natural ecosystems. To better understand and project these processes, the scientific community increasingly demands climate and weather simulations with higher spatial resolution, richer representations of physical processes, and large ensembles under multiple scenarios. Consequently, the complexity and scale of numerical models have expanded dramatically, driving exponential growth in data volumes. Global-scale simulations now routinely generate from tens to hundreds of petabytes of output (Balaji et al., 2018; Overpeck et al., 2011), placing unprecedented pressure on storage and analysis capacities. The rapid rise in data production poses significant challenges for existing storage infrastructures, constraining long-term archiving, data sharing, reproducibility of analyses, and the efficiency of post-processing workflows. These challenges are further exacerbated by storage systems and network bandwidths that have not kept pace with the rapid gains in computational performance (Prein et al., 2015).

These storage constraints are not unique to global simulations. In regional models, although they focus on smaller spatial domains, data storage management is still an inevitable challenge as spatiotemporal resolution increases. The Weather Research and Forecasting (WRF) model (Skamarock et al., 2019), a widely used regional modeling system with a large international user base across academia, government agencies, private forecasting services, and independent researchers (Powers et al., 2017), exemplifies this issue. High-resolution WRF simulations generate from several to hundreds of terabytes of output from a single project (Prein et al., 2015). Such datasets are not only central to advancing scientific understanding but are also increasingly used to support infrastructure planning, disaster preparedness, and policy development (Akbar et al., 2013; Jam-Jalloh et al., 2024; Young et al., 2025; Zhang et al., 2025). At the same time, the rapid adoption of machine learning in atmospheric science has amplified data-handling demands. WRF outputs are increasingly used as training datasets (Abdulla et al., 2022; Waqas et al., 2025), and the large size and high temporal density of these simulation archives can pose practical challenges for data preprocessing and training workflows. These combined pressures highlight the urgent need for effective data compression techniques that can substantially reduce data storage requirements.

A variety of compression techniques applicable to atmospheric model archives have been developed to alleviate the rapidly growing storage demands of numerical simulations. Lossless algorithms alone preserve bitwise reproducibility but generally achieve only a modest compression ratio for floating-point geophysical fields because the high entropy of mantissa bits limits compressibility (Poppick et al., 2020). To address this limitation, combining precision reduction with lossless compression has emerged as a widely explored strategy for improving storage efficiency while attempting to preserve scientific fidelity. Within this paradigm, many approaches focus on manipulating the least significant bits of floating-point data. Early approaches such as bit shaving (Zender, 2016) reduce precision by zeroing trailing mantissa bits, which can introduce systematic bias. Zender (2016) subsequently introduced bit grooming, a statistically robust quantization approach that alternates bit shaving and bit setting of trailing IEEE mantissa bits, thereby preserving the mean in expectation while improving compressibility. Building on this line of work, Delaunay et al. (2019) introduced the Digit Rounding algorithm, which bridges decimal precision control and binary representation. Compared to bit grooming, Digit Rounding optimizes the allocation of mantissa bits required, thereby improving compression efficiency while retaining controlled numerical precision. In contrast to hybrid methods like Digit Rounding, alternative approaches regulate precision purely within the decimal domain. For example, decimal significant-digit rounding (Walters and Wong, 2023) directly constrains the number of significant digits, yielding a highly transparent and interpretable approach to precision reduction.

Alongside algorithmic developments, some studies have expanded the evaluation of precision reduction beyond simple error statistics toward broader notions of statistical and structural fidelity. Baker et al. (2016) proposed an ensemble-based framework to assess whether compressed datasets remain statistically indistinguishable from internal climate variability, while Baker et al. (2019) emphasized the importance of evaluating structural integrity in compressed climate data. Silver and Zender (2017) quantified the compression–error trade-off for gridded datasets, demonstrating that carefully designed precision reduction can remove substantial false precision with limited impact on scientific conclusions. Complementary technical investigations have examined how data representation and codec behavior influence achievable compression ratios within netCDF and HDF data formats (Delaunay et al., 2019; Underwood et al., 2022). Recently, Klöwer et al. (2021) reframed atmospheric data compression from an information-theoretic perspective, demonstrating that the intrinsic precision requirements of atmospheric variables vary substantially and that a large fraction of stored floating-point precision is redundant. In parallel, machine-learning-based compression methods have emerged that exploit nonlinear data manifolds to achieve very high compression ratios (Huang and Hoefler, 2023; Han et al., 2024; Mirowski et al., 2024).

Despite these advancements, most existing studies primarily consider precision reduction as a post-processing operation applied to static datasets or model outputs. Comparatively less attention has been paid to how precision reduction applied to time-varying model inputs propagates through nonlinear numerical integrations. A fundamental distinction exists between the precision reduction of input data and that of output data. Perturbations introduced into the input forcings may be amplified through nonlinear model physics, potentially influencing simulated trajectories and downstream diagnostics. Such responses are unlikely to be spatially or temporally uniform, as sensitivity to perturbations depends on the prevailing dynamical and thermodynamic regime.

Crucially, the extent to which precision reduction applied to model inputs and outputs propagates into downstream diagnostic analyses remains largely unquantified. While the fidelity of fundamental thermodynamic and kinematic fields (e.g., temperature and wind) must be systematically verified, this uncertainty becomes particularly critical in downstream diagnostic studies of precipitation, which is output as a cumulative quantity and exhibits intermittent and highly skewed characteristics. Given the disproportionate societal and economic impacts of extreme precipitation (Seneviratne et al., 2021; Davenport et al., 2021), it is therefore essential to assess whether precision reduction introduces artificial biases in extreme-event diagnostics, such as inflated dry-area coverage or altered peak precipitation intensities, that exceed expected analysis uncertainty.

Motivated by these gaps, this study investigates decimal significant-digit rounding within a complete WRF workflow. Using a one-year simulation, we systematically evaluate the distinct physical consequences of applying precision reduction to both time-varying forcing inputs and static outputs. Our assessment spans fundamental meteorological fields and the full precipitation spectrum. Ultimately, this comprehensive assessment aims to provide the modeling community with actionable guidelines for implementing safe and efficient precision reduction strategies. The remainder of this paper is organized as follows: Sect. 2 describes the WRF model configuration, the decimal significant-digit rounding algorithm, and the evaluation framework applied to both time-varying input forcings and model outputs, including metrics designed to assess spatial structural fidelity and precipitation diagnostics. Section 3 presents the compression performance and examines the distinct physical consequences of input versus output precision reduction. It further analyzes variable-specific, regional, and seasonal sensitivities across core meteorological variables and precipitation ranging from zero-value preservation to ETCCDI-based extreme precipitation indices. Section 4 synthesizes these findings to provide practical guidelines and broader perspectives. Section 5 summarizes the main conclusions.

## 2 Methodology

### 2.1 Model Configuration and Data Flow

The year 2016 was chosen as the simulation period because it recorded the highest number of billion-dollar flood disasters and the second-largest economic loss in the United States since 2000 according to the NOAA National Centers for Environmental Information (NCEI) (NCEI, 2025). These factors make 2016 an ideal test benchmark for assessing model performance under different precision reduction configurations, particularly with respect to the preservation of extreme precipitation events. Furthermore, the contiguous United States (CONUS) domain was chosen because it encompasses diverse climatological regions and benefits from dense observational networks, providing a robust basis for model assessment.

Simulations were conducted using the WRF model version 4.4.1 over the CONUS domain at a 12 km horizontal spatial resolution with 35 vertical layers. To prevent systematic model drift during the long-term integration, the four-dimensional data assimilation (FDDA) via grid-nudging was employed within the WRF framework, utilizing the North American Mesoscale (NAM) analysis, produced operationally by the National Centers for Environmental Prediction (NCEP). In model settings, weak nudging coefficients (1.0 × 10^−4^ s^−1^ for wind and temperature, and 1.0 × 10^−5^ s^−1^ for moisture) were deliberately selected for the free troposphere. This configuration acts as a large-scale constraint to anchor the synoptic circulation while granting the model sufficient degrees of freedom to generate free-evolving, high-resolution mesoscale features. Furthermore, atmospheric nudging was explicitly disabled within the planetary boundary layer and at the surface to avoid suppressing near-surface thermodynamic variability. Because the boundary-layer and surface atmospheric fields evolved entirely according to model physics, subsequent evaluations of the WRF outputs against independent observational station data remain scientifically valid and uncontaminated by the direct assimilation process.

To mitigate long-term model drift in deep soil temperature and moisture, we employed the Pleim-Xiu land surface model combined with indirect soil nudging, which specifically requires the continuous ingestion of two-dimensional surface analysis fields (wrfsfdda). Coupled with the three-dimensional atmospheric fields (wrffdda) utilized for upperair nudging, the model is forced to ingest massive datasets every 3 h. The sheer volume of these high-resolution input arrays significantly exacerbates the overall storage and data management burden. Consequently, these massive time-varying input files represent a critical target for our proposed precision reduction. Table 1 summarizes the physical parameterization schemes used in the WRF simulations.

The WRF modeling system operates through a structured input–integration–output workflow. To establish dynamical consistency prior to the evaluation period, the annual simulation for 2016 was preceded by a 2 d spin-up initialized on 30 December 2015. The annual integration was conducted in consecutive daily segments, with each day initialized from a model restart file (wrfrst) generated at the conclusion of the previous day. These restart files provide the three-dimensional atmospheric and land-surface state required for seamless temporal continuity. In addition to daily initialization, the model evolution was continuously constrained by prescribed time-varying external forcing fields, including atmospheric and surface analysis nudging (wrffdda, wrfsfdda), lateral boundary condition tendencies (wrfbdy), and lower-boundary updates such as sea surface temperature (wrflowinp).

Because these time-varying inputs function as continuous external constraints, precision reduction introduced at this stage may propagate through the model integration and interact with nonlinear physical processes. In contrast, model outputs (wrfout) contain near-surface variables, three-dimensional atmospheric states, cloud microphysical quantities and energy fluxes, representing the prognostic results of the completed simulation. Post-processing modifications to these output files do not influence the meteorological evolution within the simulation itself. Recognizing this distinction is important for interpreting the impacts of precision reduction applied at different stages.

### 2.2 Decimal Significant-Digit Rounding

The input and output data of the WRF model are stored in single-precision (float32) format, which supports a numerical precision of approximately 7 significant digits. To control the precision of such single-precision netCDF datasets, this study implements a decimal significant-digit rounding method following Walters and Wong (2023). This scheme regulates numerical precision by retaining a user-specified number of significant digits. The rounding procedure was carried out using a dedicated Fortran routine based on internal character formatting: each floating-point value is first converted into a string in scientific notation using a dynamically specified Fortran format descriptor (i.e., E*w.d*, where E denotes the exponential scientific notation, the total width *w* = *n* + 7, and the number of decimal places *d = n*; here, *n* denotes the targeted number of significant digits to retain), and the formatted string is subsequently read back into floating-point representation. The precision-reduction method implemented in this study operates directly on the floating-point variables, except latitude and longitude, in the netCDF data part, which are the standard format used by WRF for both input forcing and model outputs. The rounding procedure modifies only the numerical values stored in the variable arrays and does not affect the netCDF header, metadata, or structural attributes.

The deliberate adoption of this decimal-based approach, instead of direct binary mantissa manipulation, is motivated by the following considerations: decimal significant-digit rounding enables users to intuitively understand and verify the exact numerical modifications applied to the data, thereby lowering the adoption barrier for practical modeling workflows. Nevertheless, we acknowledge the inherent compression advantages of bit-level operations. As demonstrated by Delaunay et al. (2019), algorithms that map decimal precision requirements to the binary mantissa can maximize sequences of consecutive zero bits, thus generating data patterns with higher compressibility.

Furthermore, traditional decimal rounding (rounding half away from zero), which is natively handled by Fortran, may theoretically introduce systematic biases compared to IEEE 754 round-to-nearest, ties-to-even rules (IEEE, 1985). We conducted a statistical evaluation and found that exact tie cases are rare when we practically apply the method of rounding to significant digits. Across all examined core variables (e.g. surface meteorological field and precipitation, each comprising over 1.4 billion data points), exact tie situations occurred at low frequencies: approximately 0.003 %–0.005 % for retaining 3 significant digits, 0.03 %–0.04 % for 4 significant digits, and 0.2 %–0.4 % for 5 significant digits. Because over 99.6 % of values are rounded identically under both approaches, the practical impact of rounding-mode selection on the datasets analyzed herein is negligible.

Given that single-precision format inherently provides approximately at most 7 significant digits, reducing precision to 6 digits yields only marginal compression gains. Conversely, retaining only 1 or 2 significant digits would severely degrade key variables such as temperature and pressure, rendering such configurations physically unrealistic. As a result, this study conservatively focuses on retaining 3–5 significant digits. The range of 3–5 retained significant digits is not intended to represent a universal optimal precision level for each variable but rather to provide a practical evaluation window for single-precision WRF data. Establishing strict, variable-specific theoretical precision limits would require information-theoretic evaluations (Klöwer et al., 2021).

To provide an intuitive visualization of the rounding results, Table 2 contrasts the original full-precision values against those retained at 5, 4, and 3 significant digits for key near-surface variables at a single grid point and time step: 2 m temperature (*T*_2_), 2 m specific humidity (*Q*_2_), and 10 m wind components (*U*_10_ and *V*_10_).

**Figure 1.**
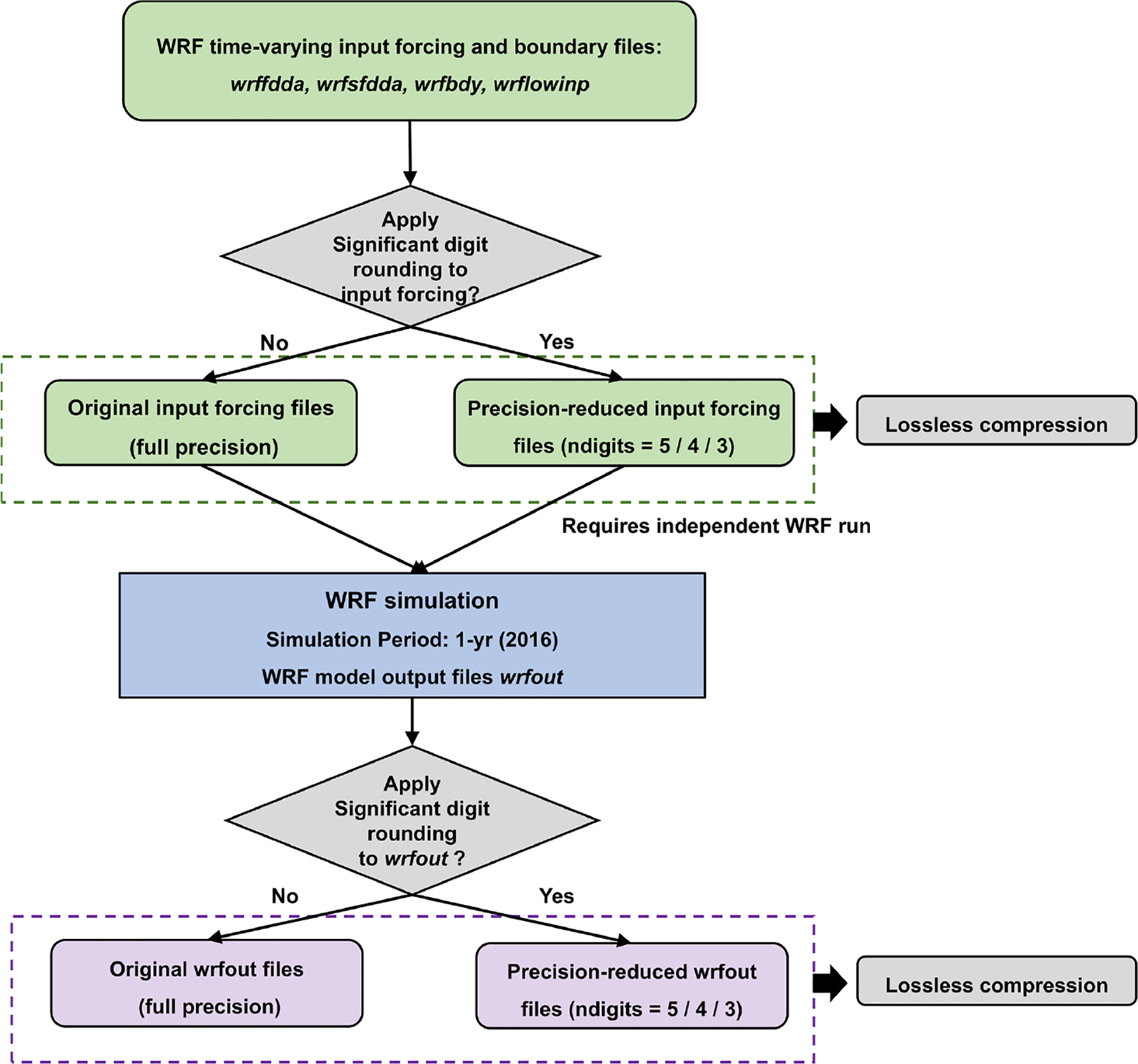
Schematic illustration of the experimental workflow for WRF data precision reduction and lossless compression.

The systematic integration of this rounding framework into the overall experimental design is summarized in Fig. 1. The schematic depicts the three core workflow stages: preprocessing on input with the precision-reduction tool, model integration with full-precision input and precision-reduction input, and post-processing on model integration result, wrfout file, with the precision-reduction tool. Lossless compression will be applied to all input and output. For input precision reduction configurations, the rounding procedure was applied during the preprocessing stage, exclusively targeting the time-varying forcing files (wrffdda, wrfsfdda, wrfbdy, and wrflowinp). Because these files define the dynamic external constraints on the model, evaluating each input precision level necessitated a fully independent WRF simulation to capture the non-linear propagation of rounding errors. Conversely, for output precision reduction, rounding was applied to the final wrfout files after simulation completion. As a purely static post-processing operation, output precision can be flexibly adjusted without requiring computationally expensive model re-runs. In total, this systematic design yielded 15 distinct precision reduction configurations (Table 3).

It is important to note that the decimal significant-digit rounding procedure effectively reduces the information entropy of the datasets, but it does not alter the predefined 32-bit storage allocation of the floating-point arrays. To translate this enhanced compressibility into tangible storage savings, lossless compression was subsequently applied to all 15 precision reduction configurations. In this study, gzip and bzip2 were selected as the primary external compressors for evaluation, given their widespread availability in operational high-performance computing (HPC) environments and compatibility with existing modeling workflows. To account for format-integrated compression, we also evaluated the internal zlib compression of netCDF, which is commonly used within the netCDF/HDF5 framework and enables direct metadata access without full data decompression. In addition, to align with contemporary data storage practices, we included targeted evaluations using the newer-generation codec Zstandard (Zstd). A comparative assessment of compression performance across these compressors is presented in Sect. 3.1.

### 2.3 Observational Datasets and Climate Regional Subdivision

**Figure 2.**
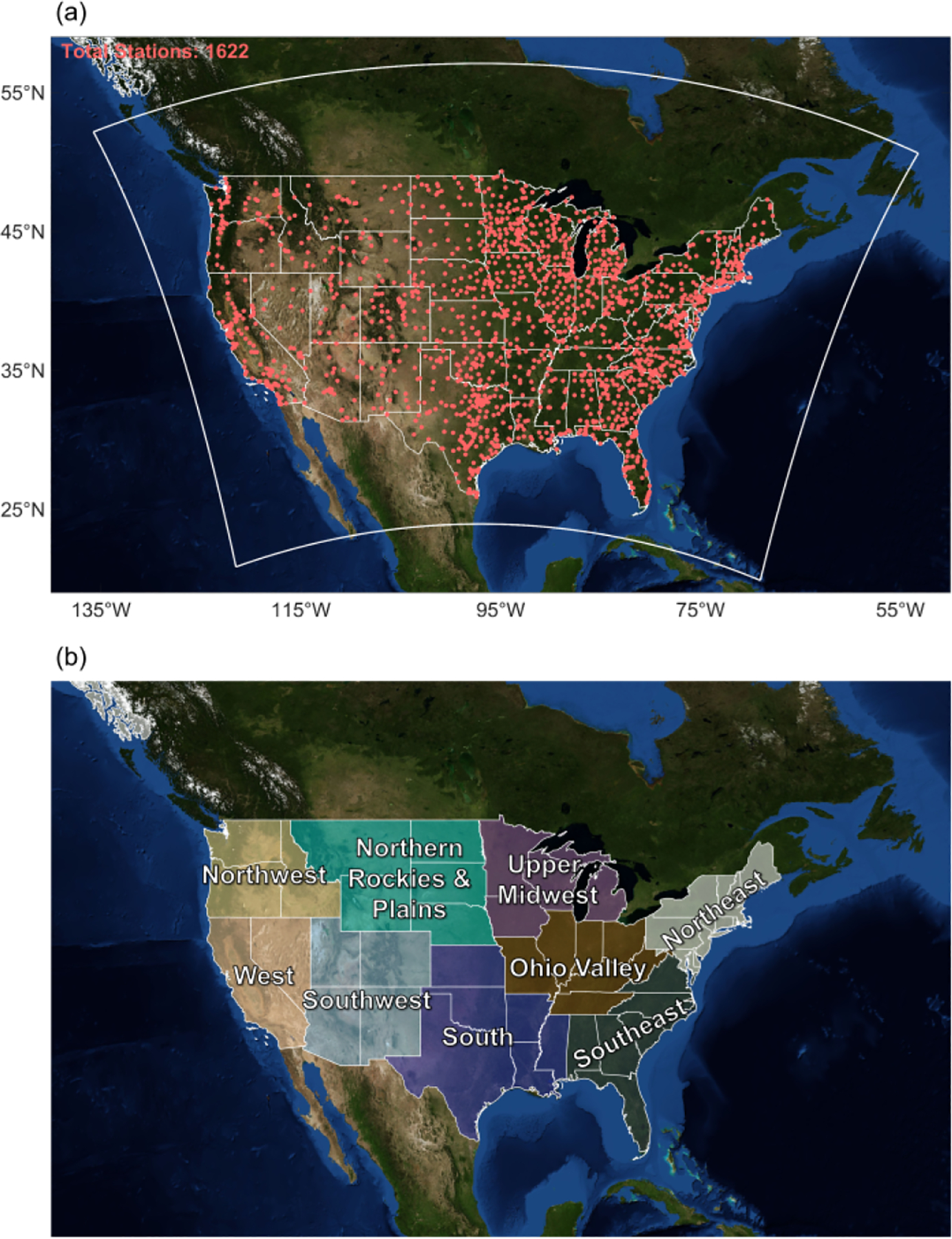
Spatial coverage of surface observational datasets, WRF model domain, and regional divisions: **(a)** locations of NCDC surface meteorological stations (red dots) across CONUS domain; the white box outlines the WRF simulation domain; **(b)** delineation of nine climatologically coherent subregions. Base map imagery from NASA Worldview, Earth Observing System Data and Information System (EOSDIS).

To quantitatively evaluate model performance, we employed observational datasets of surface meteorology and precipitation for the year 2016 within the CONUS domain. Hourly near-surface meteorological variables, including *T*_2_, relative humidity (RH), and wind speed (WS10), were obtained from the National Climatic Data Center (NCDC). To ensure data reliability and spatial representativeness, a two-step quality control procedure was applied. First, only stations with valid records for at least 330 d in 2016 and with less than 5 % missing data were retained. Second, to mitigate representativeness errors caused by complex terrain, we excluded stations with an elevation difference exceeding 100 m from their corresponding WRF grid cell. These criteria yielded a total of 1622 valid stations (Fig. 2a), providing dense spatial coverage across diverse climatic and physiographic regions. To spatially align the point-based measurements with the gridded model output, a nearest-neighbor interpolation scheme was applied, assigning each station to the WRF grid point with the smallest Euclidean distance in the latitude–longitude space. Despite the inherent representativeness limitations typical of point-to-grid regional climate validation, this extensive 1622-station network provides a robust benchmark for assessing the model’s meteorological fidelity at both regional and CONUS-wide scales.

For precipitation validation, we used the Global Precipitation Measurement (GPM) Integrated Multi-satellite Retrievals for GPM (IMERG) Final Precipitation L3 product (Version 07) with a uniform spatial resolution of 0.1° × 0.1°. This dataset was processed across two distinct temporal scales to accommodate different evaluation objectives. First, to establish a climatological baseline, daily IMERG data for the period 2001–2015 (Huffman et al., 2023b) were used to compute the 95th and 99th percentile precipitation thresholds. These historical thresholds were subsequently applied to define and calculate extreme precipitation indices for the study period. Second, for the primary 2016 evaluation, IMERG half-hourly data (Huffman et al., 2023a) were temporally aggregated to an hourly resolution to align with the WRF model output frequency. These hourly records were further aggregated to daily intervals for the computation of the aforementioned extreme precipitation indices. Finally, all IMERG datasets were bilinear interpolated onto the WRF model grid prior to statistical evaluation to ensure spatial consistency between the satellite retrievals and the model domain.

To capture the spatial heterogeneity of errors induced by precision reduction, the CONUS domain was subdivided into nine climatologically coherent regions following Karl and Koss (1984): Northwest, West, Northern Rockies and Plains, Southwest, Upper Midwest, Ohio Valley, South, Northeast, and Southeast (Fig. 2b).

### 2.4 Evaluation Metrics

To systematically evaluate the impacts of precision reduction on model fidelity, we adopted a multi-level assessment framework designed to capture statistical consistency, maximum numerical deviations, spatial structure, and highly skewed precipitation characteristics.

First, overall statistical agreement between precision-reduced simulations and observational datasets was evaluated using two standard community metrics: the Root Mean Square Error (RMSE) and the Pearson correlation coefficient (*R*). These metrics quantify the overall magnitude of simulation errors and the strength of the linear association with the observations, respectively. These metrics are defined as follows:

(1) $$\text{RMSE}=\sqrt{\frac{1}{N}\sum_{i=1}^{N}{\left(M_{i}-O_{i}\right)}^{2}}$$

(2) $$R=\frac{\sum_{i=1}^{N}\left(M_{i}-\overset{-}{M}\right)\left(O_{i}-\overset{-}{O}\right)}{\sqrt{\sum_{i=1}^{N}{\left(M_{i}-\overset{-}{M}\right)}^{2}\sum_{i=1}^{N}{\left(O_{i}-\overset{-}{O}\right)}^{2}}}$$

where *M*_*i*_ and *O*_*i*_ denote the evaluated values (precision-reduced configurations) and the observations, respectively, and overbars represent their corresponding means. For station-based validation against NCDC observations, *N* corresponds to the number of valid station–hour pairs across all stations. For comparisons against gridded satellite products, *N* corresponds to the total number of grid–time pairs within the evaluated domain.

Second, to evaluate the impacts of precision reduction on both local numerical accuracy and spatial structures, we analyzed point-wise deviations together with spatial similarity metrics. At the grid scale, we computed the absolute grid-scale deviation (AD), defined as the absolute difference between the precision-reduced configurations and the full-precision baseline simulation WRF_bl at each grid point and hourly time step. Because point-wise metrics alone cannot capture changes in the spatial organization of meteorological fields, we additionally employed the Structural Similarity Index Measure (SSIM) (Wang et al., 2004; Baker et al., 2019; Klöwer et al., 2021). This metric is particularly important for evaluating simulations driven by precision-reduced inputs, where small numerical perturbations may propagate through nonlinear dynamics and alter evolving mesoscale structures. Unlike AD, which measures local differences, SSIM quantifies the preservation of large-scale spatial patterns and structural textures. For a full-precision reference spatial window *x* and the corresponding window *y* from precision-reduced configurations, the SSIM is calculated as:

(3) $$\text{SSIM}\left(x,y\right)=\frac{\left(2\mu_{x}\mu_{y}+C_{1}\right)\left(2\sigma_{\text{xy}}+C_{2}\right)}{\left(\mu_{x}^{2}+\mu_{y}^{2}+C_{1}\right)\left(\sigma_{x}^{2}+\sigma_{y}^{2}+C_{2}\right)}$$

where *μ*_*x*_ and *μ*_*y*_ are the local means, $\sigma_{x}^{2}$ and $\sigma_{y}^{2}$ are the local variances, and *σ*_*xy*_ is the local covariance between the two fields. The stabilization constants *C*_1_ and *C*_2_ are scaled by the dynamic range of the specific meteorological variable being evaluated to accommodate diverse atmospheric fields. SSIM values range from 0 to 1, with unity indicating perfect structural similarity. Structural similarity was computed using an 11 × 11 Gaussian weighting window sliding across the domain. Given the 12 km horizontal grid spacing, this corresponds to a spatial footprint of approximately 130 km, enabling assessment of mesoscale structural consistency. To avoid artificial inflation of scores caused by large, structurally uniform dry regions, a standard wet-day mask with a threshold of 0.1 mm h^−1^ was applied prior to the calculation of precipitation SSIM.

Finally, the evaluation of precipitation for both the lower-tail thresholds that control the occurrence of light rainfall and the behavior of upper-tail extreme events is necessary given the highly skewed distribution of precipitation fields. Moreover, because total precipitation in WRF is represented as the cumulative sum of grid-resolved (RAINNC) and parameterized convective (RAINC) components, it exhibits a compounded sensitivity to numerical precision loss.

To assess structural fidelity at the lower bound of the precipitation spectrum, we first performed a categorical verification for hourly precipitation, using a wet–dry threshold of 0.1 mm h^−1^. For each precision-reduced configuration, grid cells were classified relative to WRF_bl. Based on this comparison, several categorical verification metrics were calculated (Roebber, 2009), including the Probability of Detection (POD), False Alarm Ratio (FAR), Critical Success Index (CSI), and Frequency Bias (Bias). In addition, a Zero Preservation Ratio (ZPR) was introduced to quantify the fraction of dry grid cells that remain correctly classified after precision reduction. We further evaluated daily precipitation diagnostics, including eight indices recommended by the Expert Team on Climate Change Detection and Indices (ETC-CDI) (Frich et al., 2002; Nastos et al., 2013; Ozer and Mahamoud, 2013). These indices collectively characterize precipitation intensity, frequency, and persistence. Additionally, to explicitly quantify any systematic overestimation or underestimation of these extreme precipitation magnitudes resulting after precision reduction, we computed the Normalized Mean Bias (NMB). To ensure climatological representativeness, percentile-based thresholds used by indices such as R95p_days and R99p_days were derived from the 2001–2015 daily GPM IMERG baseline dataset. The definitions and units of all precipitation diagnostics used in this study, including both the categorical verification metrics and the extreme precipitation indices described above, are summarized in Table 4. Note that unlike the standard ETCCDI definitions which calculate R95p and R99p as accumulated precipitation amounts, this study utilizes R95p_days and R99p_days to represent the frequency of extreme events.

## 3 Results

### 3.1 Compression Efficiency Analysis

To quantify the impact of decimal significant-digit rounding on data compressibility, we analyze compression performance using two complementary metrics. First, we evaluate the additional compression gain relative to lossless compression alone, which isolates the contribution of rounding. Second, we assess the overall storage reduction relative to the original uncompressed datasets to provide an intuitive measure of practical data savings. All calculations are benchmarked against a full-precision baseline (stored in the standard 32-bit single-precision floating-point format) comprising 837.0 GB of uncompressed input forcings and 7395.8 GB of uncompressed output data.

**Figure 3.**
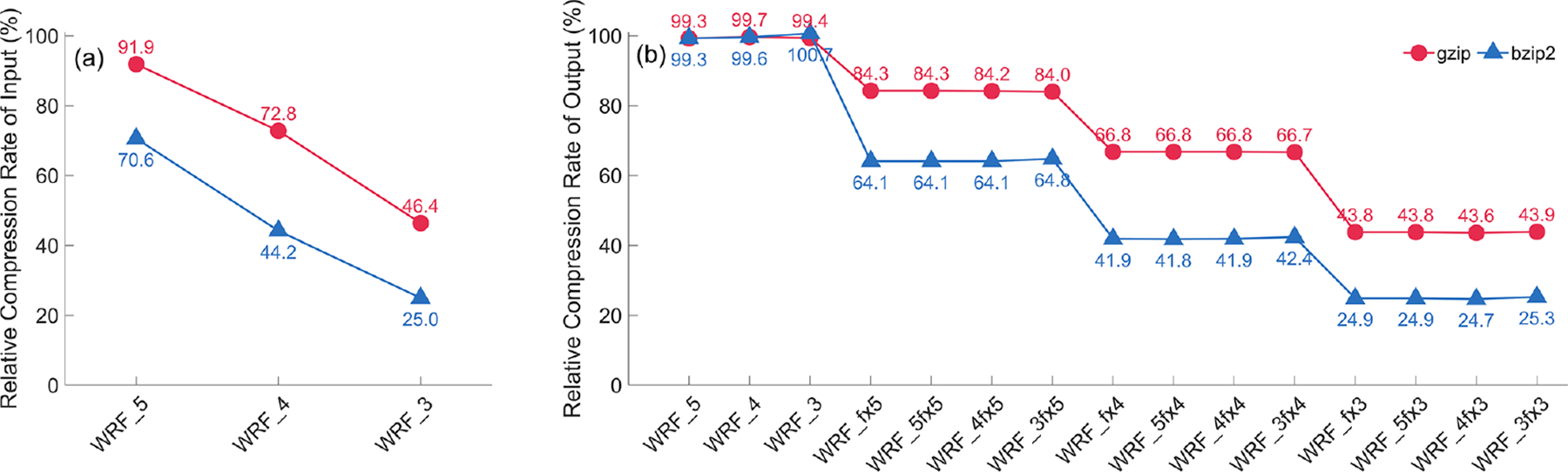
Relative compression ratios of **(a)** input data and **(b)** output data across diverse precision reduction configurations.

As summarized in Table 5 and illustrated in Fig. 3, data compressibility improves progressively with more aggressive precision reduction. Here, the relative compression ratio is defined as the compressed size of the precision-reduced configurations divided by the compressed size of the full-precision baseline. This metric isolates the additional space savings attributable specifically to decimal significant-digit rounding. For input datasets (Fig. 3a), the relative compression ratio under gzip decreases from 91.9 % (retaining 5 significant digits) to 46.4 % (retaining 3 significant digits), while bzip2 achieves a reduction from 70.6 % to 25.0 %. For output datasets (Fig. 3b), the ratio decreases from 84 % to 44 % under gzip, and from 64 % to 25 % under bzip2. As physically expected, the final compressed volume of the output is dictated by the precision applied during the output post-processing stage; varying the input precision exerts no immediate influence on the final compressed wrfout file size. On average, bzip2 outperforms gzip by 15–30 percentage points across all precision-reduced configurations (Table 5). From a practical storage perspective, when retaining 3 significant digits and using bzip2, the size of the compressed input and output datasets is approximately one-quarter of that achieved with lossless compression alone. Specifically, the compressed datasets account for 18.5 % and 7.5 % of the original uncompressed data volume, respectively.

We additionally evaluated format-native netCDF internal compression (utilizing zlib deflation) alongside external compressors, including gzip, bzip2, and the newer-generation, multi-threaded Zstd codec. Because our precision-reduction procedure strictly modifies only the floating-point data arrays while preserving the original file structure, the resulting datasets remain fully compatible with the internal compression functionality of netCDF. This native integration provides an important operational advantage: because netCDF internal compression applies strictly to data variables, the metadata headers remain uncompressed and directly accessible. This enables rapid metadata inspection and lazy loading without triggering decompression of the underlying data variables, resulting in effectively zero decompression time in our benchmarks (reported as 0.00 s). Benchmark experiments were conducted for both input and output datasets across different precision-reduction configurations, and the compression performance for a representative 5 d WRF input dataset and a 1 d WRF output dataset is summarized in Tables 6 and 7, respectively. Throughout the following discussion, specific compressor configurations are denoted by parentheses indicating their settings; for instance, Zstd (19,8) denotes Zstandard applied at compression level 19 using 8 threads, and zlib (9) refers to zlib deflation at compression level 9.

The benchmark results reveal a clear trade-off between storage efficiency and computational cost, and this trade-off differs between full-precision and precision-reduced datasets. For the raw full-precision data, the best storage reduction is achieved by high-level Zstd, which slightly outperforms both bzip2 and zlib. For example, for the WRF output file (Table 7), Zstd at level 19 reduces the file to 29.78 % of its original size, compared with 30.34 % for zlib (9), 30.46 % for bzip2, and 30.66 % for gzip. A similar but smaller advantage is found for the full-precision input file (Table 6), where Zstd (19,8) reduces the file to 74.14 % of its original size, compared with 74.80 % for zlib (9) and 75.11 % for bzip2. In addition, low-level Zstd provides markedly faster compression than bzip2 and gzip, while multi-threaded Zstd (19,8) recovers much of the runtime cost associated with high compression levels.

Once significant-digit rounding is applied, however, the relative ranking changes substantially. Across all tested precision-reduced configurations, bzip2 consistently achieves the smallest final file sizes, while Zstd remains the strongest practical alternative, and zlib and gzip occupy an intermediate position. For example, for the WRF input file with 3 significant digits retained (Table 6), bzip2 reduces the dataset to 18.56 % of its original size, compared with 25.47 % for Zstd (19,8), 33.63 % for zlib (9), and 34.95 % for gzip. For the WRF output file with 3 significant digits retained (Table 7), the corresponding values are 7.65 % for bzip2, 10.16 % for Zstd (19,8), 12.96 % for zlib (9), and 13.58 % for gzip. Thus, after precision reduction, the practical ordering is generally bzip2 > Zstd > zlib ≈ gzip in terms of compression ratio. Notably, zlib level 1 provides a balanced native option, but maximum native compression at zlib level 9 becomes increasingly expensive as precision is reduced, with compression times rising sharply for heavily rounded datasets (e.g., 1289 s for the input with 3 significant digits retained and 1125 s for the output with 3 significant digits retained) without proportional storage benefits.

These differences reflect both the underlying compression mechanisms and their algorithmic implementations. Within the LZ77-based family (Zstd, gzip, zlib), Zstd employs more efficient entropy coding (Finite State Entropy, FSE), which explains its superior performance on raw full-precision data (Collet and Kucherawy, 2021). However, decimal rounding transforms long repeated byte sequences into quasirepetitive, imperfect patterns primarily reflected in the lower-order mantissa bits, reducing the effectiveness of dictionary-based matching. For zlib, this limitation is further amplified by its operation within HDF5 chunk boundaries, which limits the exploitation of redundancy beyond local chunks, and by deeper dictionary hash-chain traversals at high compression levels. As a result, the algorithm incurs substantial computational overhead while searching for longer matching sequences among near-matches, without proportional gains in compression efficiency. In contrast, compressors such as bzip2 process data in larger blocks and employ the Burrows–Wheeler Transform (Burrows and Wheeler, 1994). By globally reordering data prior to entropy encoding, BWT effectively clusters scattered, non-contiguous bit patterns introduced by precision reduction, enabling more efficient exploitation of redundancy than LZ77-based methods. Consequently, bzip2 consistently achieves the highest compression ratios for these rounded datasets.

In conclusion, consistent with previous studies (Zender, 2016; Silver and Zender, 2017), precision reduction substantially improves subsequent standard lossless compression, which is also reflected in our results obtained with the decimal significant-digit rounding method. The substantial reduction in storage footprint achieved by reducing input and output precision underscores the potential of this coupled precision reduction and lossless compression strategy within the WRF workflow. However, while these physical storage benefits are substantial, the scientific consequences of precision reduction remain a critical concern.

### 3.2 Observational Validation of Macroscopic Impacts

**Figure 4.**
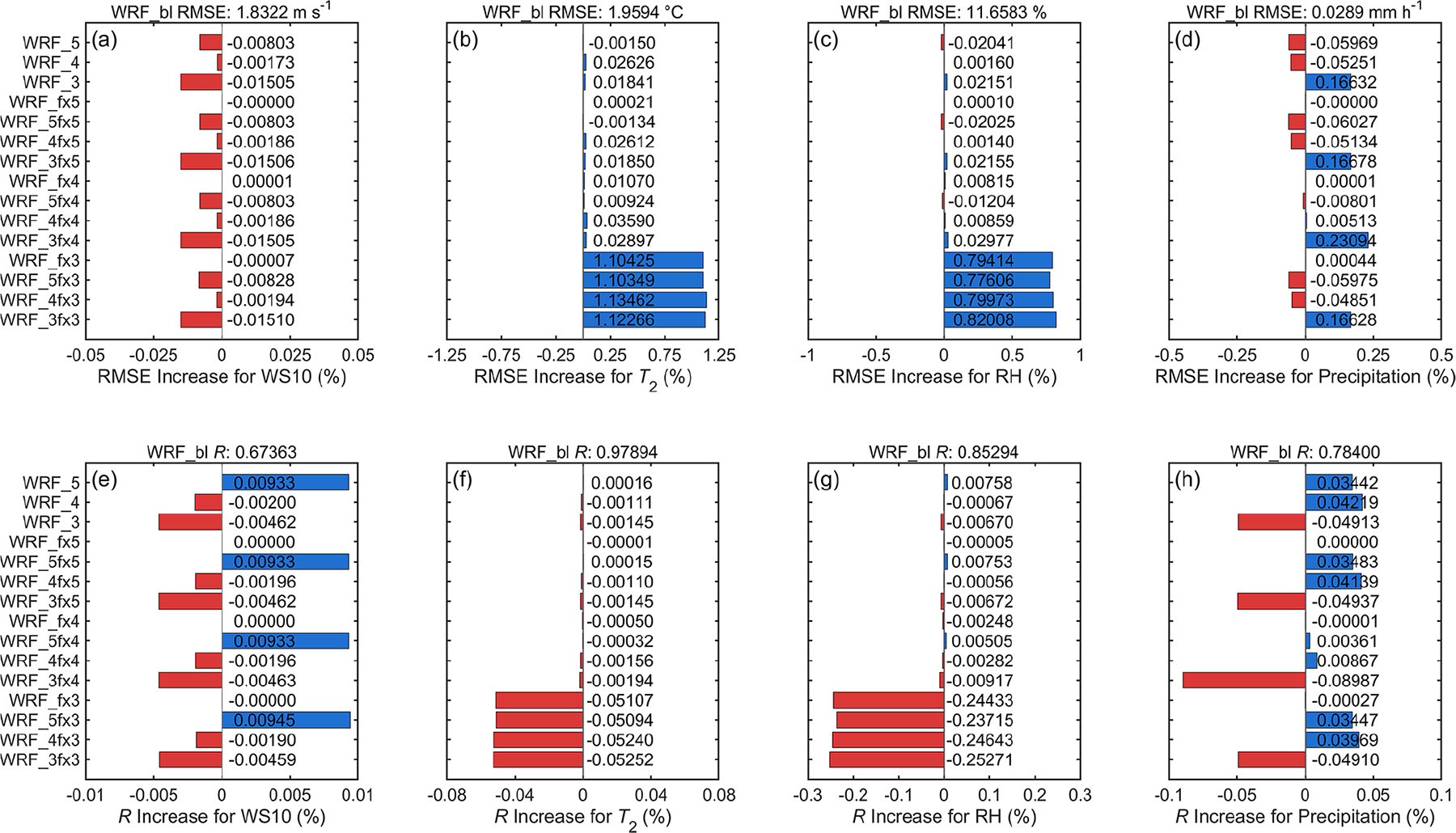
Relative changes with regard to WRF_bl, in RMSE **(a–d)** and correlation coefficient *R* **(e–h)** for wind speed, temperature, relative humidity, and precipitation across all cases.

While improvements in data compressibility are important, they must be weighed against potential degradation in scientific fidelity. To ascertain whether precision reduction undermines the model’s capacity to simulate real-world processes, we first validated the simulations against observational datasets. Figure 4 presents the relative changes in RMSE and correlation coefficient *R* for WS10, *T*_2_, RH, and precipitation, evaluated against observational references. The relative change is defined as the percentage difference in these metrics between each precision-reduced configuration and the WRF_bl, with both configurations independently evaluated against the same observational datasets. For precipitation, RMSE and *R* were calculated using the spatially averaged regional time series. For all other variables, these statistics were first computed at individual stations and subsequently averaged across all sites.

For WS10, the error growth is predominantly driven by input precision reduction, exhibiting a larger and nonlinear response compared to output-only precision reduction. This suggests that wind dynamics are more sensitive to perturbations in input forcings. However, this variable exhibits the smallest relative error variation among all variables evaluated. This indicates that despite its high sensitivity to input perturbations, the overall magnitude of the induced bias remains strictly bounded. In contrast, thermodynamical fields such as *T*_2_ and RH exhibit different behavior. Reducing the output of *T*_2_ to 3 significant digits produces larger changes relative to other configurations, with RMSE increasing by about 1.1 % and *R* decreasing by about 0.05 %. This behavior is largely a numerical artifact stemming from the Kelvin scale used in WRF outputs, where retaining 3 significant digits effectively removes all sub-degree decimal precision. Importantly, the pronounced sensitivity of RH, when output precision is reduced to 3 significant digits, is intrinsically tied to these temperature deviations. Governed by the non-linear Clausius–Clapeyron relationship, numerical artifacts from temperature directly propagate into saturation vapor pressure calculations, rendering the observed RH deviations largely secondary thermodynamic artifacts. Additionally, direct observational validation of absolute moisture was precluded: deriving specific humidity or mixing ratio from dew point temperature requires concurrent pressure data, yet rigorous quality control of these pressure records limited the available data to merely a few hundred valid stations domain-wide, rendering the network statistically insufficient for robust spatial verification. For precipitation, while reducing the input precision to 3 significant digits leads to larger deviations relative to other configurations, the absolute degradation remains minimal. This demonstrates that although precipitation is strongly modulated by upstream dynamical fields, its bulk macroscopic fidelity is still resilient.

**Figure 5.**
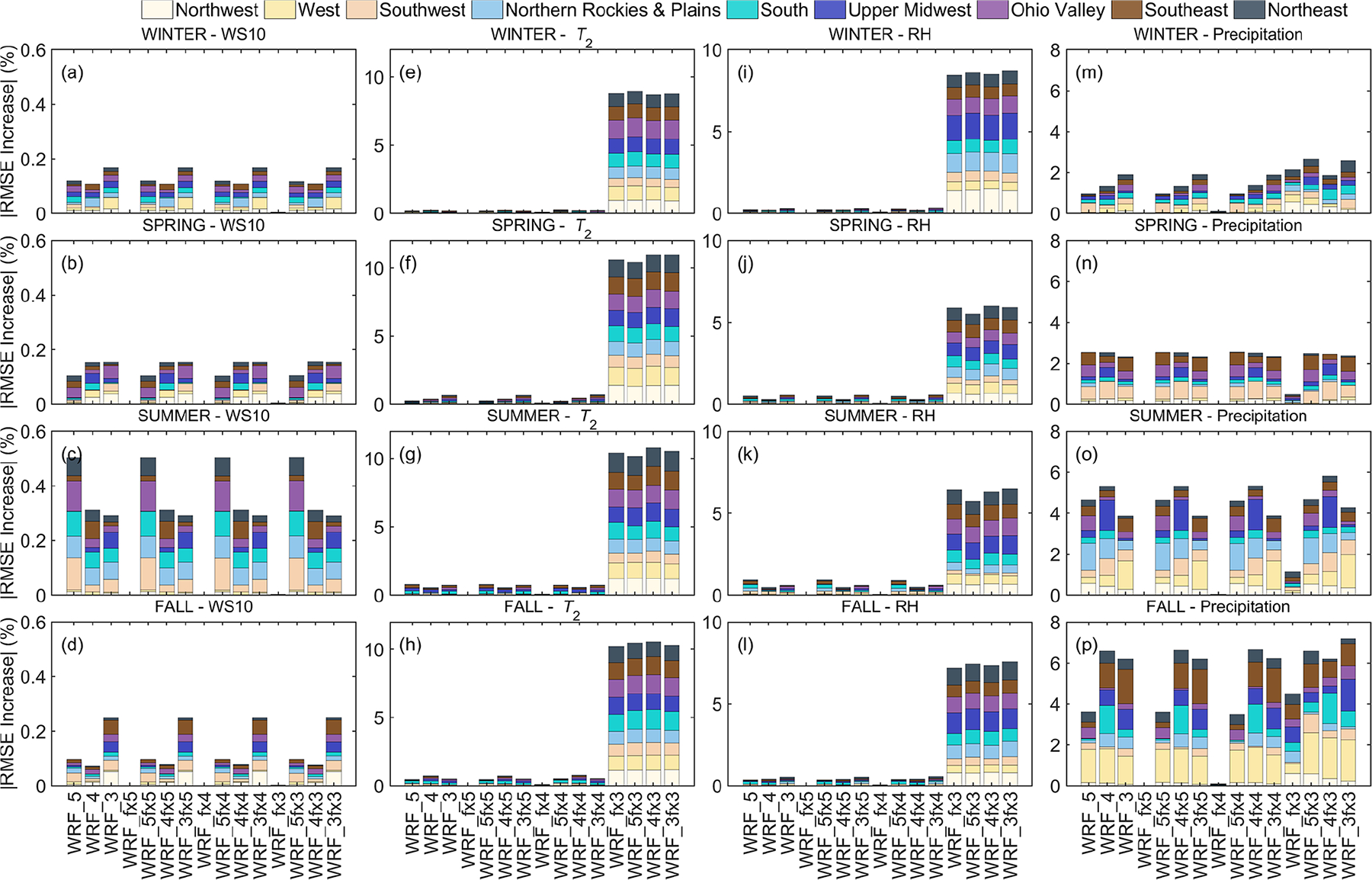
Magnitude of seasonal and regional relative changes in RMSE for meteorological variables across 15 precision-reduced configurations. Stacked bars illustrate the regional breakdown of | RMSE increase | relative to the WRF_bl across nine climate regions. Note that the total bar heights serve solely to visualize relative regional contributions and do not represent a mathematically aggregated domain-wide RMSE percentage. Results are presented separately for **(a–d)** wind speed, **(e–h)** temperature, **(i–l)** relative humidity, and **(m–p)** precipitation.

Further decomposition of these errors highlights substantial regional and seasonal heterogeneity, as shown in Fig. 5. Figure 5 illustrates the absolute relative change in RMSE for other configurations relative to WRF_bl across nine climate regions and four seasons. When output-only precision is reduced to 5 or 4 significant digits, the resulting error increments are negligible for all variables across regions and seasons. By contrast, reducing the output to 3 digits exerts a discernible influence: while WS10 remains largely stable, *T*_2_ and RH show consistent relative RMSE increases across all regions and seasons, and precipitation displays noticeable degradations.

Regarding WS10, it remains the most robust variable, with relative RMSE increments remaining below 0.1 % across nearly all regions and seasons. The small error peaks observed are primarily associated with input precision reduction and exhibit modest regional and seasonal signatures, such as slight increases over the Southwest and Ohio Valley during summer. This behavior suggests that wind fields are comparatively tolerant to precision reduction and can accommodate more aggressive output precision reduction. Across configurations with 3-digit output precision, the relative RMSE increment of *T*_2_ is approximately 1.2 % with spatial coherence. A similar pattern is also observed for RH, although with minor regional variability, likely attributed to its nonlinear dependence on temperature via the Clausius–Clapeyron relationship. Precipitation emerges as the most heterogeneous field: relative RMSE increases are largest in fall, followed by summer, and smaller in winter and spring, with regional contributions shifting seasonally from the Upper Midwest, West, Southwest, and Northern Rockies and Plains in summer to the West, Southeast, Upper Midwest, and South in fall. Compared with these predictable output responses, precision-reduced input exerts a less systematic influence, producing non-monotonic and regionally heterogeneous biases, particularly evident in wind speed and precipitation. Together, these results highlight a fundamental distinction in how precision loss influences model fidelity: retaining 4–5 significant digits in model outputs generally preserves meteorological consistency, whereas more aggressive precision reduction introduces variable-dependent sensitivities. Thermodynamic variables such as *T*_2_ and RH exhibit increased susceptibility when retaining 3 significant digits, while WS10 remains comparatively insensitive. In contrast, input perturbations interact with the model’s nonlinear dynamics, leading to spatially heterogeneous and temporally evolving deviations in dynamical and diagnostic fields.

### 3.3 Maximum Deviations and Structural Fidelity

While domain-averaged metrics provide a robust macroscopic assessment of simulation fidelity, they may obscure localized extremes and spatial distortions. To more comprehensively evaluate model reliability under different precision-reduced configurations, we therefore examined grid-scale maximum AD and the domain-wide SSIM.

As summarized in Table 8, precision reduction applied to model inputs generally produces substantially larger maximum AD than that of output. In some cases, these deviations appear extreme. For example, under the WRF_4 configuration, *T*_2_ exhibits grid-point maximum AD approaching ~ 21.45 K. Similarly, under WRF_3, hourly precipitation reaches a maximum AD of ~ 145.45 mm h^−1^. Despite these seemingly severe local deviations, the annual minimum SSIM for *T*_2_ remains above 0.97 under WRF_4. The annual minimum SSIM for precipitation is approximately 0.86 under WRF_3, but at the exact moment when precipitation deviation reaches its peak, the SSIM approaches 0.99. This indicates that the occurrence of large point-wise deviations is not necessarily accompanied by structural breakdown of the simulated fields. The corresponding results for the remaining configurations in Table 8 are provided in Table S1 in the Supplement.

**Figure 6.**
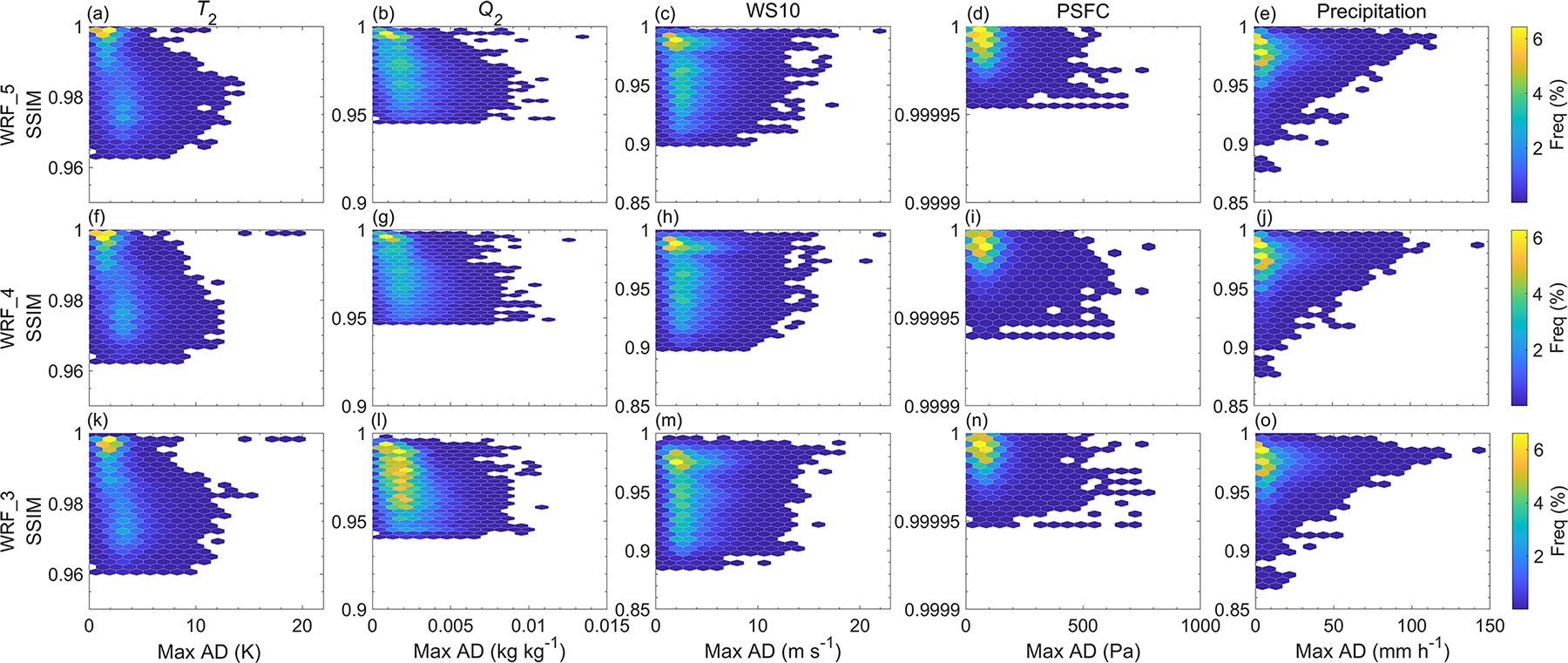
Hexbin density plots showing the grid-scale maximum AD and the corresponding SSIM. Results are presented for five variables (*T*_2_, *Q*_2_, WS10, PSFC, and precipitation) with input precision reduced to 5, 4, and 3 significant digits, respectively.

To further clarify this behavior, we examined the relationship between the maximum AD at each grid point and the SSIM computed at the corresponding time when that maximum deviation occurs (Fig. 6). The results reveal a clear decoupling between grid-point maximum deviation and structural fidelity under input precision-reduced configurations. This suggests that large point-wise deviations can arise from spatial or temporal phase shifts of otherwise coherent meteorological features, rather than indicating a fundamental degradation of the simulated atmospheric structure. In other words, under input perturbations, the simulated system maintains morphological integrity yet may have undergone spatial or temporal displacement. Snapshot comparisons at the time of maximum AD for *T*_2_ and precipitation (see Figs. S1 and S2 in the Supplement) provide further visual evidence supporting this interpretation. Structural sensitivity also exhibits strong variable dependence. Large-scale continuous fields such as PSFC remain highly robust: even under WRF_3, the 1st percentile SSIM exceeds 0.9997. In contrast, precipitation as an intermittent diagnostic variable shows more pronounced degradation.

**Figure 7.**
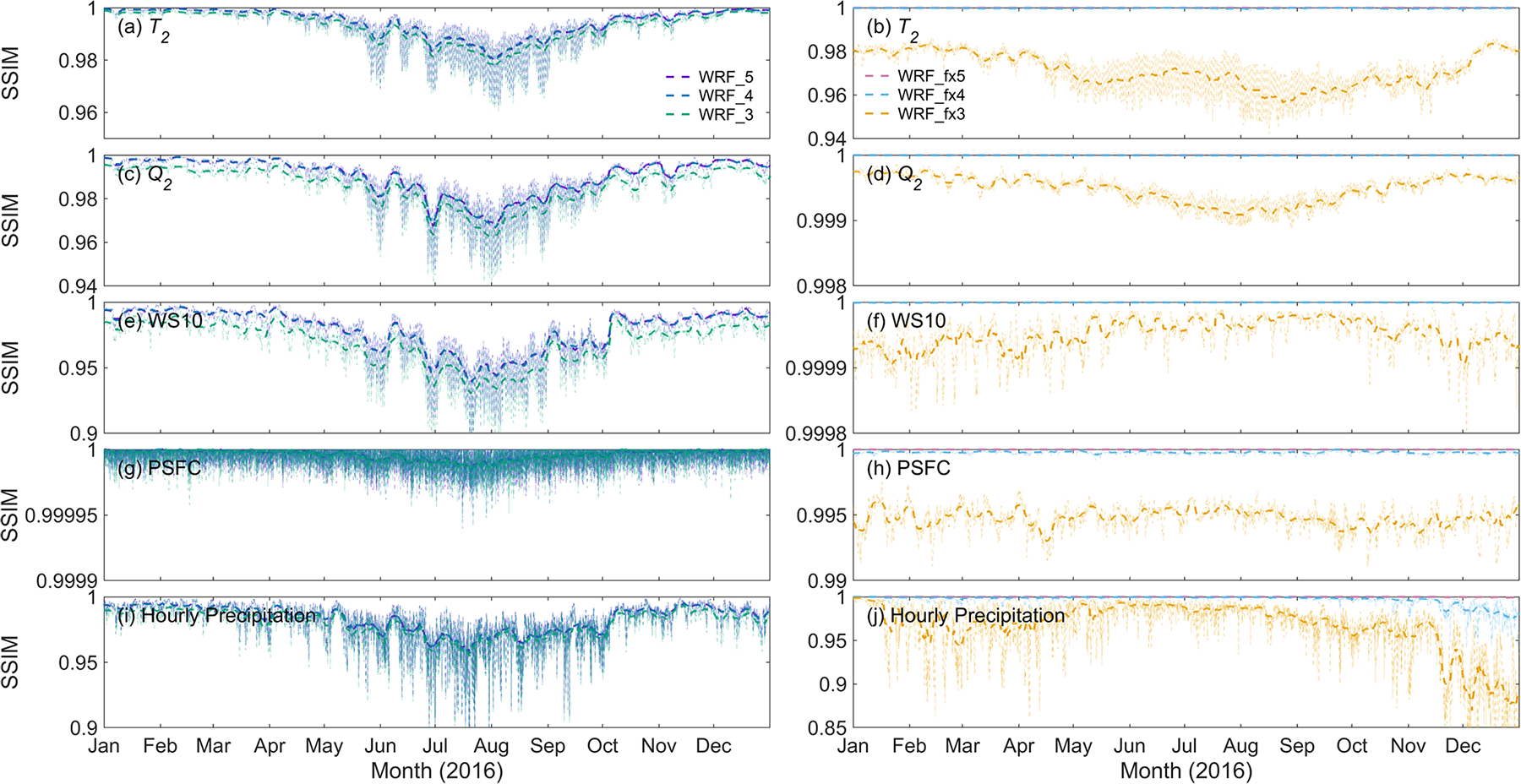
Time series of SSIM for five variables (*T*_2_, *Q*_2_, WS10, PSFC, and precipitation) under different precision-reduced configurations. The fine dotted lines represent the original value of hourly SSIM, and the thick dashed lines represent the rolling median of hourly SSIM.

The temporal evolution of SSIM (Fig. 7) further highlights two fundamentally distinct error modes associated with precision reduction. As illustrated in the left column of Fig. 7, input precision reduction exhibits a consistent seasonal variability characterized by a “U-shaped” depression in SSIM from May to September. During dynamically active periods, nonlinear atmospheric processes amplify small perturbations introduced through time-varying forcing fields. In contrast, output-only precision-reduced configurations (right column of Fig. 7) produce a markedly different temporal signature. The degradation in SSIM appears as a steady, step-like decline with minimal seasonal influence, consistent with static mathematical rounding artifacts rather than dynamically evolving divergence. This is particularly evident in Table 8, as exemplified by PSFC, where the maximum AD under WRF_fx5, WRF_fx4, and WRF_fx3 is strictly bounded at 5, 50, and 500 Pa, respectively.

Output-only precision reduction exposes an inherent sensitivity in cumulative variables when hourly precipitation is derived through temporal differencing. Under WRF_fx3, this sensitivity manifests as a time-dependent degradation of structural fidelity in hourly precipitation fields. From late January to April, the rolling median SSIM decreased to approximately 0.95, as 72 % of grid cells exceeded ~ 100 mm for RAINNC and 34 % for RAINC (Fig. S3a, b). At this magnitude, retaining 3 significant digits for both RAINC and RAINNC variables introduces a rounding error of approximately 2 mm h^−1^, which readily overwhelms weak stratiform precipitation signals.

Interestingly, although individual grid cells cross the 1000 mm accumulation threshold much earlier in the simulation, by March for RAINNC and June for RAINC, the SSIM collapse occurs much later. This apparent temporal inconsistency can be explained by the combined effects of spatial coverage and precipitation regime. In the early months of the simulation, only a small fraction of grid cells exceeds this threshold (~ 1.3 % for RAINNC in May, with RAINC being negligible), limiting its impact. From June to October, although the affected fraction increases (RAINNC ~ 6.8 %, RAINC ~ 16.9 %), intense convective rainfall produces large hourly increments to partially mask the quantization noise. This effectively restores the signal-to-noise ratio and partially masks the numerical artifacts. However, a sharp SSIM decline occurs post-November (SSIM rolling median ~ 0.88). By this time, the fraction of grid cells exceeding 1000 mm further increases (RAINNC ~ 10 %, RAINC ~ 18 %) (Fig. S3c, d). At this > 1000 mm baseline, the 3-significant-digit constraint significantly expands the quantization interval, potentially generating artificial jumps approaching ~ 20 mm h^−1^. Meanwhile, the precipitation regime reverts to being dominated by weaker stratiform precipitation. Under these conditions, the massive quantization noise entirely dominates the physical signal, rendering the true precipitation gradients largely unresolvable. A similar, though weaker, behavior is observed under WRF_fx4. While retaining 4 significant digits preserves near-perfect structural similarity (SSIM approaches 1) through most of the simulation period, a similar structural degradation paradigm emerges post-November.

These findings provide important methodological implications for balancing storage efficiency and scientific validity. In the context of this study, one practical strategy is to retain 4 significant digits prior to November and increase the precision to 5 significant digits thereafter, thereby protecting the weak stratiform precipitation signals that dominate during the later stages of the simulation. More generally, if scientific accuracy is prioritized, a dynamic precision reduction configuration can be adopted for cumulative precipitation variables. In such a scheme, the compression algorithm monitors accumulated precipitation and initially retains 4 significant digits, automatically upgrading to 5 significant digits once cumulative precipitation exceeds 1000 mm at the regional average or even grid scale.

Finally, the appropriate threshold for SSIM warrants further investigation, particularly when accounting for model resolution and the sources of precision reduction. Baker et al. (2019) adopted SSIM thresholds exceeding 0.99995 for coarser-resolution datasets (~ 100 km). At the 12 km resolution considered here, SSIM becomes more sensitive to the spatial displacement of fine mesoscale structures. Importantly, tolerance for SSIM degradation should be interpreted differently for input and output precision reduction. When precision reduction is applied to time-varying input forcings, the moderate reductions in SSIM may reflect physically interpretable displacement rather than structural collapse. In contrast, output-only precision reduction introduces purely static numerical artifacts without interacting with model dynamics. Consequently, SSIM degradation in this pathway reflects artificial structural distortion rather than physical displacement. For this reason, the tolerance for SSIM should remain substantially stricter, typically requiring values exceeding 0.999 to ensure that numerical artifacts do not corrupt spatial structures.

### 3.4 Impacts on Precipitation Diagnostics: Zero-Value Preservation and Extreme Precipitation Indices

While the SSIM analysis reveals how these numerical artifacts alter the spatial structure of precipitation fields, an equally important question is how distortions introduced by precision reduction in cumulative precipitation fields propagate into downstream scientific diagnostics. Precipitation exhibits a highly skewed spatial and temporal distribution, characterized by two distinct and sensitive regimes: a widespread lower tail dominated by zero or near-zero values, and a rare but high-impact upper tail driven by extreme rainfall events. Both regimes are particularly vulnerable to numerical artifacts introduced by precision reduction. To quantify these impacts, the following section evaluates how precision-reduced configurations affect precipitation diagnostics, including zero-value preservation and extreme precipitation indices.

We first evaluate the preservation of the precipitation occurrence spectrum, specifically focusing on the zero-value boundary (using the 0.1 mm h^−1^ wet-day threshold). Standard contingency metrics, including ZPR, POD, FAR, CSI, and Bias, are employed to diagnose how different compression configurations alter the fundamental wet/dry morphology (Table 9).

For input-only precision reduction configurations, the metrics indicate that the overall precipitation spectrum is largely preserved. The Frequency Bias remains close to unity (e.g., 0.99942 for WRF_3), confirming that the total precipitation area is conserved. Furthermore, the nearly symmetric error rates, where the moderate FAR (7.84 %–8.72 %) closely balances the corresponding miss rates (1 – POD), together with a stable CSI (~ 0.84) indicate that input precision reduction induces spatial phase shifts in precipitation systems rather than systematically disrupting their structure. In contrast, aggressive output precision reduction introduces substantial numerical distortions that directly erode the bottom end of the precipitation spectrum, most prominently in the WRF_fx3 configuration. Under this configuration, the POD drops sharply to 71.94 %, indicating that approximately 28 % of valid light precipitation events are artificially truncated to zero due to the loss of significance in the arithmetic of cumulative precipitation variables. As a result, a pronounced systematic dry bias emerges (Bias ≈ 0.77).

Interestingly, WRF_fx3 exhibits an apparently higher ZPR (99.12 %) and a lower FAR (6.90 %) compared with WRF_fx4 (98.5 % and 8.98 %, respectively). However, this does not indicate improved structural fidelity. Instead, it reveals that WRF_fx3 suppresses both numerical noise (reducing FAR) and genuine light stratiform precipitation (reducing POD), effectively converting them into widespread nonphysical dry regions.

The structural degradation previously indicated by the late-stage decline in SSIM is further corroborated by the temporal evolution of precipitation occurrence metrics under the WRF_fx4 configuration (Fig. S4). At the domain scale, the ZPR remains high (> 97 % throughout the year), largely reflecting the overwhelming dominance of climatologically dry background grids. However, metrics that evaluate performance specifically within precipitation events (CSI, POD, and FAR) reveal a pronounced late-stage deterioration. Consistent with the SSIM decline observed in November, the CSI decreases from its summer peak (~ 0.89) to 0.769 by December. This degradation reflects a dual numerical distortion associated with the expanding accumulation baseline. As the quantization step increases during November and December, the FAR rises substantially (reaching 11.4 %), indicating the growing occurrence of spurious drizzle signals introduced by rounding artifacts. At the same time, the POD declines to 85.4 %, implying that approximately 15 % of genuine winter stratiform precipitation events are artificially truncated to zero. The Frequency Bias further highlights this shift in behavior. While the system exhibits a slight wet bias in spring, it gradually transitions to a systematic dry bias by December (Bias = 0.964). The simultaneous increase in false alarms and the loss of weak precipitation events progressively disrupt the spatial consistency of precipitation structures, definitively explaining the late-stage SSIM collapse.

The analyses above focus on the lower boundary of the precipitation spectrum, where precision reduction primarily affects the occurrence of light rainfall and the preservation of dry conditions. However, precipitation diagnostics are also strongly influenced by the opposite end of the distribution. To assess these upper-tail characteristics of precipitation, we next evaluate a suite of extreme precipitation indices. For clarity, the indices are grouped into two categories. The first category consists of percentile-based and maximum-based extreme precipitation indices (R95p_days, R99p_days, Rx1_day, Rx5_day), which emphasize the upper tail of the daily precipitation distribution and the magnitude of rare extreme events. The second category includes precipitation frequency and accumulation indices (R10mm_days, PRCPTOT, wet_days, SDII), which describe total precipitation amounts, mean intensity, and the occurrence of moderate wet events.

**Figure 8.**
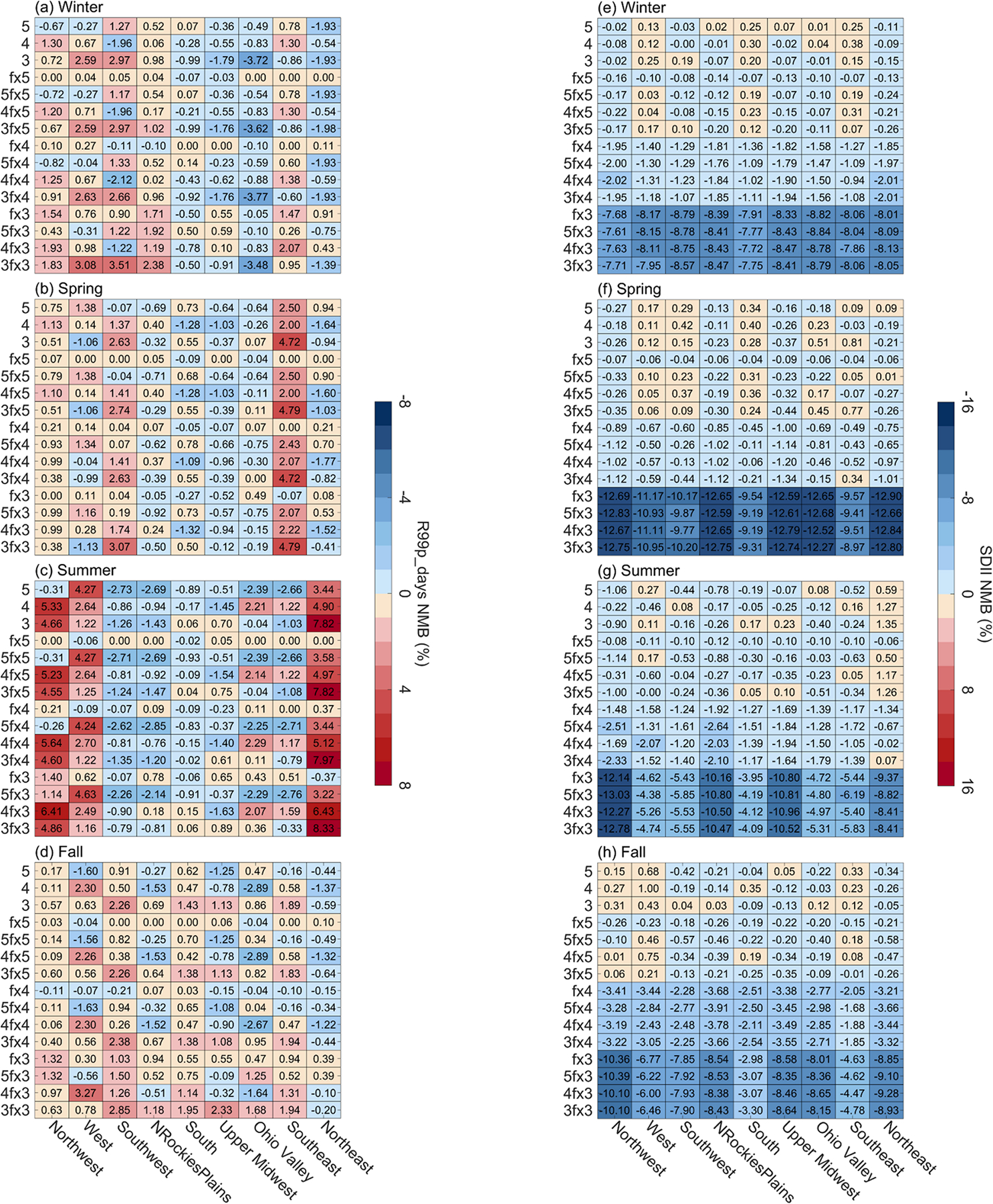
Seasonal and regional NMB of R99p_days **(a–d)** and SDII **(e–h)** relative to WRF_bl, shown for different precision-reduced configurations across nine climate regions and four seasons.

Figure 8 focuses on two representative diagnostics, R99p_days and SDII, while results for the remaining six indices are provided in Figs. S5–S10. These extreme precipitation metrics further corroborate that distinct precision reduction pathways (input vs. output pathways) exert impacts on different components of the precipitation spectrum. For the percentile-based index R99p_days, NMB fluctuates within ±3 % during winter, spring, and fall, while it amplifies in summer to a maximum bias of 8.33 % (Fig. 8a–d). This pronounced summer overestimation is highly localized, particularly in the Northwest and Northeast, and is fundamentally driven by input precision reduction (retaining 3 significant digits). In the conditionally unstable summer atmosphere, input perturbations can artificially induce and expand spurious convective initiation, thereby systematically inflating the R99p_days count. In contrast, output-only precision reduction produces comparatively small (<1.5 %) and near-linear response from WRF_fx5 to WRF_fx3. Once combined with the precision-reduced input, however, the response becomes distinctly nonlinear. Consequently, percentile-based extreme precipitation metrics are particularly sensitive to the spatial perturbations associated with input precision reduction. In contrast, SDII exhibits a different behavior. Negative biases dominate across most regions and seasons, and these biases are primarily governed by output precision configuration (Fig. 8e–h). The WRF_fx4 configuration yields consistent reductions of approximately −1 % to −3 %, while the more aggressive WRF_fx3 configuration produces substantially larger decreases ranging from −3 % to 13 %.

**Figure 9.**
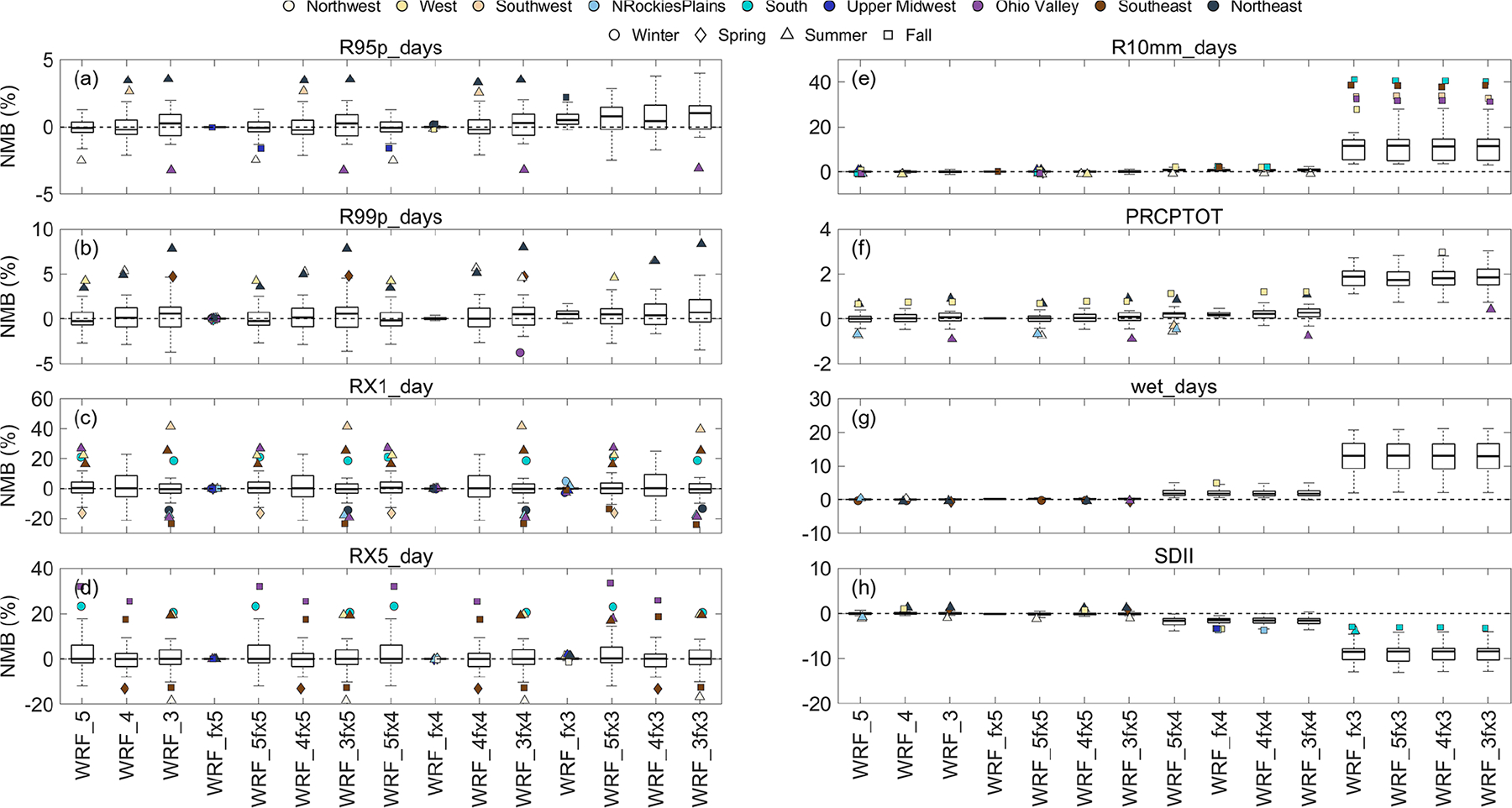
Box plots showing the distribution of NMB values for extreme precipitation indices across climate regions and seasons. Each box spans the interquartile range (IQR; 25th–75th percentiles), with the median shown as a horizontal line. Whiskers extend to 1.5× IQR, and outliers are plotted as colored shapes corresponding to different climate regions and seasons. Dashed horizontal lines indicate zero bias.

**Figure 10.**
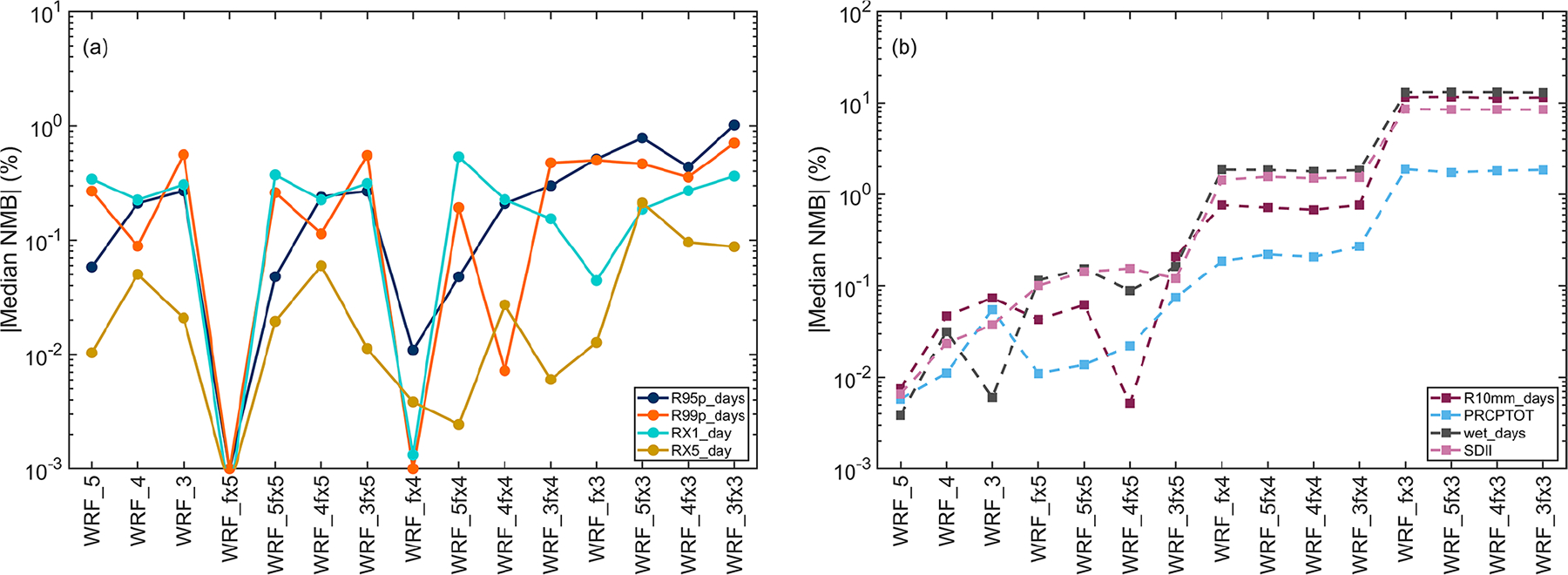
The absolute value of the median NMB of extreme precipitation indices across 15 precision reduction configurations and nine climate regions relative to the WRF_bl. (a) shows the indices for extreme precipitation events (R95p_days, R99p_days, Rx1_day, Rx5_day), while panel (b) displays the indices for precipitation frequency and intensity (R10mm_days, PRCPTOT, wet_days, SDII).

The distinct mechanism underlying these responses becomes clearer when examining the full set of eight indices using box plots (Fig. 9). For the extreme-intensity group (R95p_days, R99p_days, Rx1_day, Rx5_day), output-only precision reduction configurations keep tight interquartile ranges, whereas input precision reduction configurations generate broader spreads and numerous outliers due to spatial phase displacement. In contrast, the frequency and accumulation group (R10mm_days, PRCPTOT, wet_days, SDII) is primarily governed by quantization artifacts associated with output precision reduction. When outputs are retained to 3 significant digits under large accumulation baselines, coarse quantization steps (e.g., artificial increments approaching ~ 20 mm) can spuriously push daily totals across fixed thresholds such as 1 or 10 mm. This effect artificially increases the occurrence of wet days, producing positive biases in both R10mm_days and wet_days. Because SDII is defined as PRCPTOT divided by wet_days, the artificial expansion of the denominator directly induces the systematic negative bias observed in SDII. To isolate the systematic error trajectories from the regional spatial noise observed in the box plots, Fig. 10 synthesizes the absolute values of the median NMB across all 15 configurations, reinforcing these conclusions.

Figures 8–10 collectively demonstrate that downstream diagnostic outcomes at the extreme tail of the precipitation spectrum are inherently more sensitive to input precision reduction, with their biases exhibiting highly nonlinear characteristics and marked regional and seasonal divergence. While aggressive output precision reduction (retaining 3 significant digits) can induce error magnitudes comparable to those from input precision reduction, the resulting biases remain more concentrated statistically. In contrast, precipitation frequency and accumulation indices follow fundamentally distinct degradation pathways: as output precision decreases, they are strictly dominated by systematic biases, a structural distortion that is severely amplified when retaining 3 significant digits. Finally, it is critical to recognize the inherent limitations in interpreting the seasonal divergences of precipitation. Given that output precision reduction acts on a continuously accumulating precipitation field, this temporal classification can occasionally conflate true atmospheric seasonality with the mathematical artifacts of cumulative growth. For example, as illustrated in Fig. 9, retaining only 3 significant digits for precipitation output generates anomalously high R10mm_days frequencies heavily concentrated in autumn (September–November). This apparent ’seasonal’ peak is, in reality, a numerical illusion: by autumn, the annual accumulated precipitation has grown to a massive background magnitude where 3-digit retention enforces excessively coarse quantization steps. Consequently, temporal differencing at this late stage of integration artificially and frequently triggers the 10 mm daily exceedance threshold. However, it is worth noting that for precipitation intensity indices (e.g., the left column in Fig. 9), the pronounced summer anomalies are indeed physically meaningful. This limitation profoundly underscores the inherent challenges of conducting traditional seasonal analyses on cumulative variables under precision-reduced configurations.

## 4 Practical Guidelines and Broader Perspectives for Precision Reduction Configuration

Building on the analyses presented in Sect. 3, we translate the numerical and physical findings into practical guidance for precision reduction within the WRF modeling workflow. The results reveal that different precision reduction pathways introduce fundamentally different error mechanisms, implying that compression strategies must be selected according to downstream scientific objectives and the physical nature of the variables involved.

### 4.1 Implications of Input Precision Reduction

Precision reduction applied to time-varying model inputs introduces perturbations that interact with nonlinear atmospheric dynamics during model integration. These perturbations propagate through the simulation and may alter the precise spatial and temporal positioning of weather systems. As demonstrated in Sect. 3.3, such perturbations can generate large grid-scale maximum AD. However, the simultaneously high SSIM values indicate that the overall morphology of the simulated atmospheric systems remains largely preserved. Consequently, the resulting errors primarily manifest as pronounced maximum AD driven by local spatial or temporal phase shifts, accompanied by a background of widespread, disordered weak perturbations, rather than a fundamental structural collapse of the simulated fields. Therefore, input precision reduction may be acceptable for applications that prioritize large-scale statistics or aggregated diagnostics, where exact grid-scale correspondence is not required. However, this pathway introduces intrinsic nondeterministic perturbations into the simulation, meaning that the exact location and timing of individual weather systems may shift. For impact-oriented applications requiring strict spatiotemporal consistency, such as event attribution or local hydrological analyses, this loss of deterministic correspondence may be undesirable.

In addition, the chaotic amplification of these input perturbations exhibits strong seasonal dependence. Error propagation peaks during the summer when local land-atmosphere feedback and convective processes dominate. Hypothetically, aggressive precision reduction could be applied to input forcings during less sensitive, non-summer months, while retaining full precision during the dynamically volatile summer to help constrain the cascading growth of nonlinear errors. However, the extent to which this adaptive approach would restore summer fidelity remains an open question warranting further investigation.

### 4.2 Implications of Output Precision Reduction

In contrast to the input precision reduction, output precision reduction acts as a post-processing filter. It provides a more controllable approach for impact-oriented studies requiring strict deterministic fidelity, as it ensures weather systems occur at the exact time and location simulated by the baseline physics. However, the optimal precision level is highly contingent on the intrinsic mathematical properties and computational derivations of the target variable. Based on our evaluation, several prominent typologies emerge that dictate distinct compression behaviors and require tailored precision strategies.

For fundamental state variables, compressibility is primarily governed by their numerical distributions and physical measurement limits. Atmospheric state variables exhibit markedly different compressibility characteristics determined by their numerical distributions. Dynamic variables such as WS10 are typically concentrated within a narrow numerical range, retaining 3 significant digits completely guarantees the preservation of at least one decimal place (e.g., a resolution of 0.1 m s^−1^). Therefore, applying a universal 3-digit retention for wind fields represents the most conservative compromise between storage reduction and precision, introducing negligible numerical distortion in our evaluation. Theoretically, retaining 2 significant digits could also be a viable configuration. Because 2 digits mathematically preserve single-decimal resolution for values below 10 m s^−1^, perceptible quantization errors would primarily be expected to emerge only when wind speeds exceed this threshold. Consequently, to maximize storage benefits without compromising physical fidelity, future applications could implement a magnitude-aware adaptive strategy for wind fields: dynamically toggling between 2 and 3 retained significant digits based on a 10 m s^−1^ threshold. In contrast, thermodynamic variables like *T*_2_ exhibit greater sensitivity and generally require at least 4 significant digits to prevent systematic, sub-degree information loss associated with rounding in Kelvin units. Unlike wind speed, which is subject to distinct magnitude threshold jumps (e.g., crossing the 10 m s^−1^ boundary), temperatures measured in Kelvin consistently occupy a stable, high-magnitude numerical range. Because of this stable magnitude, applying a uniform retention of 4 significant digits across the entire temperature field is entirely sufficient and structurally robust, precluding the need for dynamic precision switching. This pronounced heterogeneity in compressibility aligns profoundly with Klöwer et al. (2021), which demonstrates that a variable-specific precision reduction strategy emerges as a key paradigm for optimizing output data compression.

In stark contrast to instantaneous state variables, the WRF precipitation variable, a cumulative quantity with a highly skewed distribution, can reach thousands of millimeters annually. This continuously growing accumulation introduces fundamentally different numerical vulnerabilities, making it the primary bottleneck for precision reduction design. Specifically, retaining a fixed 3 significant digits critically widens the effective quantization interval over time (e.g., approaching ~ 20 mm h^−1^). When hourly or daily precipitation is derived through temporal differencing, this coarse quantization introduces contradictory artifacts: it suppresses very light (0.1 mm h^−1^) rainfall increments (producing a systematic dry frequency bias) while intermittently generating large, discrete step-increments, which artificially elevates the frequency of false wet_days and R10mm_days threshold exceedances. To maintain realistic precipitation statistics without indiscriminately inflating file sizes, a magnitude-aware dynamic precision strategy, scaling the retained digits according to the evolving cumulative total, emerges as a highly practical solution. Specifically, for grid cells where the accumulated precipitation remains below 1000 mm, retaining 4 significant digits is generally sufficient to preserve a scientifically viable sub-millimeter resolution. Once the accumulation surpasses the 1000 mm threshold, the precision should be dynamically increased to 5 significant digits to explicitly prevent the decimal resolution from degrading. For multi-year simulations, where total accumulations routinely exceed 10 000 mm, retaining 6 significant digits becomes strictly necessary. It is worth noting that designing a lower-tier threshold (e.g., at 100 mm) is operationally unnecessary; most simulated regions rapidly exceed this baseline shortly after initialization, rendering any lower-precision tier computationally transient and practically redundant.

Considering the specific application context, precision required for post-processed diagnostics is not universally applicable. The acceptable extent of precision reduction is ultimately determined by the relative magnitude between the physical signals that downstream analyses intend to resolve and the background state of the variable. The retained precision must be sufficient to resolve the order-of-magnitude contrast between the background state and the targeted physical perturbation, but only when resolving that perturbation is scientifically necessary. For example, atmospheric pressure typically has a background magnitude of ~ 10^5^ Pa. If a study seeks to diagnose mesoscale pressure gradients on the order of ~ 10^1^ Pa, retaining at least five significant digits becomes necessary to avoid numerical loss of information. Conversely, if the scientific objective focuses only on large-scale synoptic patterns, where variations of ~ 10^2^ Pa are sufficient to characterize the system, retaining four significant digits may already provide adequate fidelity while significantly improving storage efficiency. Following analogous reasoning, water vapor mixing ratio typically exhibits background magnitudes of ~ 10^−2^–10^−4^ kg kg^−1^, while dynamically relevant perturbations associated with moisture advection may occur at ~ 10^−5^ kg kg^−1^. Resolving such subtle signals requires at least four significant digits. However, studies concerned primarily with bulk moisture transport or large-scale moisture budgets may tolerate lower precision because these micro-scale perturbations contribute negligibly to the targeted diagnostics. Therefore, the level of precision reduction should ideally be chosen based on the requirements of the intended downstream scientific analysis, ensuring that numerical compression does not obscure the physical signals that the research aims to diagnose. When downstream applications are broad and unpredictable, as is typically the case for static reanalysis datasets distributed by institutional centers, providers are typically required to maintain conservative, high-precision baselines. Conversely, in the context of active model post-processing (e.g., targeted WRF simulations), researchers often have well-defined scientific objectives. This allows them to precisely tailor the extent of precision reduction to their specific needs, maximizing storage efficiency without compromising the physical signals of interest.

## 5 Conclusions

As high-resolution regional climate simulations generate unprecedented data volumes, combining precision reduction with lossless compression offers a promising pathway to alleviate growing storage constraints. However, the implications of this strategy within a complete regional modeling workflow remain insufficiently understood. This study systematically evaluated the impacts of decimal significant-digit rounding applied to time-varying WRF inputs, model outputs, and their combinations, assessing both fundamental meteorological variables and precipitation-related diagnostics. Through this analysis, we provide practical, workflowaware guidance for implementing precision reduction in regional climate modeling.

From a storage perspective, removing redundant numerical precision prior to compression yields substantial additional storage savings beyond conventional lossless compression. For model outputs, retaining 5 to 3 significant digits prior to bzip2 compression reduces data volumes to 19.2 %–7.5 % of their original uncompressed sizes (corresponding to 64.1 %–24.9 % relative to lossless compression alone). Comparable reductions relative to the original uncompressed dataset are achieved with Zstd (21.8 %–10.2 %), zlib (25.6 %–13.0 %), and gzip (25.4 %–13.2 %). Similarly, precision-reduced model inputs paired with bzip2 compress files to 52.4 %–18.5 % of their original volumes (corresponding to 70.6 %–25.0 % relative to lossless compression alone), while Zstd, zlib and gzip achieve 58.3 %–25.5 %, 69.1 %–33.6 % and 69.0 %–34.9 %, respectively. The overall effectiveness of precision reduction is intrinsically coupled to the choice of the backend lossless compression codec. Our findings demonstrate that for data processed via decimal significant-digit rounding, a distinct operational trade-off exists between storage efficiency and compression/decompression execution time. For long-term archival storage, where maximum compression ratio is the primary objective, bzip2 remains the preferred choice despite its higher computational cost. In contrast, for active research workflows involving frequent data access and post-processing, Zstd provides a more balanced solution, offering near-optimal compression efficiency together with substantially faster compression and decompression speeds. The internal compression zlib of netCDF represents a complementary option, particularly in environments where direct metadata accessibility and seamless format integration are required.

Beyond storage efficiency, the scientific impacts of precision reduction depend fundamentally on whether it is applied to model inputs or outputs and on the intrinsic characteristics of individual variables. Precision reduction applied to time-varying inputs introduces perturbations that interact with nonlinear model dynamics during integration. Although the large-scale atmospheric structures remain morphologically coherent, the resulting spatial phase shifts can substantially affect localized diagnostics (e.g., precipitation). Consequently, input precision reduction should generally be avoided when exact spatiotemporal correspondence is required, while it remains a viable approach for preserving aggregate statistical distributions. In contrast, output precision reduction acts as a static post-processing filter whose impacts follow predictable numerical behavior. For fundamental state variables, acceptable precision levels depend on their characteristic magnitudes: dynamic fields such as WS10 remain robust under 3 significant digits, whereas thermodynamic fields such as *T*_2_ typically require at least 4 significant digits. Cumulative variables, particularly precipitation, generally require higher precision (typically 4–5 significant digits) to prevent coarse quantization intervals from distorting derived hourly increments during temporal differencing. In addition, diagnostics derived through arithmetic combinations of multiple variables may be sensitive to numerical cancellation effects; therefore, the retained precision must remain sufficient to resolve the magnitude difference between background states and the targeted physical perturbations.

Overall, these results demonstrate that applying a uniform compression strategy across all meteorological variables is suboptimal because it inevitably sacrifices either storage efficiency or scientific fidelity. Instead, precision reduction can be implemented within a flexible, application-driven framework, which offers a practical pathway to substantially reduce data storage requirements while preserving the physical integrity of high-resolution regional climate simulations.

## Supplementary Material

Supplement1

**Supplement.** The supplement related to this article is available online at https://doi.org/10.5194/acp-26-7261-2026-supplement.

## Figures and Tables

**Table 1.** Setup of physical parameterization schemes in WRF simulation.

Physical processes	Scheme	Reference
Microphysics	Morrison double-moment	Morrison et al. (2009)
Radiation	RRTMG Shortwave and Longwave	Iacono et al. (2008)
Surface Layer	Pleim–Xiu	Pleim (2006)
Land Surface Model	Pleim–Xiu Land Surface Model	Pleim and Gilliam (2009)
Planetary Boundary Layer	Asymmetric Convection Model 2	Pleim (2007)

**Table 2.** An example of retaining different significant digits for WRF output variables.

Variables	Full precision	5-digit	4-digit	3-digit
*T*_2_ (K)	290.3516	290.35	290.4	290
*Q*_2_ (kg kg^−1^)	0.01852282	0.018523	0.01852	0.0185
*U*_10_ (m s^−1^)	0.5504633	0.55046	0.5505	0.551
*V*_10_ (m s^−1^)	−1.483089	−1.4831	−1.483	−1.48

**Table 3.** The 15 distinct precision reduction configurations evaluated in this study.

Case	Input Precision	Output Precision
WRF_fx5/fx4/fx3	Full precision	Retaining 5/4/3 significant digits
WRF_5	Retaining 5 significant digits	Full precision
WRF_5fx5/5fx4/5fx3	Retaining 5 significant digits	Retaining 5/4/3 significant digits
WRF_4	Retaining 4 significant digits	Full precision
WRF_4fx5/4fx4/4fx3	Retaining 4 significant digits	Retaining 5/4/3 significant digits
WRF_3	Retaining 3 significant digits	Full precision
WRF_3fx5/3fx4/3fx3	Retaining 3 significant digits	Retaining 5/4/3 significant digits

**Table 4.** Definitions of categorical verification metrics for hourly precipitation and daily extreme precipitation indices.

Name	Definition	Units
ZPR	Percentage of dry grids (< 0.1 mm h^−1^) in WRF_bl that remain below the threshold after precision reduction.	%
POD	Percentage of wet grids (≥ 0.1 mm h^−1^) in WRF_bl that are correctly retained after precision reduction.	%
FAR	Percentage of wet grids after precision reduction that are false alarms relative to WRF_bl.	%
CSI	A comprehensive metric for the spatial fidelity of the precipitation field, penalizing both the artificial elimination (missed events) and generation (false events) of wet events.	–
Bias	Ratio of the total number of wet grids in the precision-reduced configurations to that in WRF_bl. Values > 1 indicate an artificial inflation of precipitation spatial extent.	–
R95p_days	Number of days per year with daily precipitation exceeding the 95th percentile of wet-day amounts (≥ 1 mm), thresholds derived from the 2001–2015 baseline period.	days
R99p_days	Same as R95p_days, but for the 99th percentile threshold.	days
Rx1_day	Maximum 1 d precipitation total in a year.	mm
Rx5_day	Maximum total precipitation accumulated over any consecutive 5 d period.	mm
R10mm_days	Annual count of days with daily precipitation ≥ 10 mm.	days
PRCPTOT	Total annual precipitation from wet days.	mm
wet_days	Annual count of wet days (≥ 1 mm).	days
SDII	Simple Daily Intensity Index, calculated as PRCPTOT divided by wet_days.	mm d^−1^

**Table 5.** Storage and compression performance of input and output data across diverse precision-reduced configurations.

		gzip			bzip2		
Data Type	Configurations	Compressed size (GB)	% of Orig. Size	Relative compression ratio (%)	Compressed size (GB)	% of Orig. Size	Relative compression ratio (%)
INPUT (Original size: 837 GB)	WRF_bl	628.4	75.1	–	621.5	74.3	–
	WRF_5	577.4	69.0	91.9	438.5	52.4	70.6
	WRF_4	457.5	54.7	72.8	274.5	32.8	44.2
	WRF_3	291.7	34.9	46.4	155.1	18.5	25.0
OUTPUT (Original size: 7395.8 GB)	WRF_bl	2229.5	30.1	–	2212.6	29.9	–
	WRF_5	2214.0	29.9	99.3	2196.6	29.7	99.3
	WRF_4	2223.3	30.0	99.7	2203.8	29.8	99.6
	WRF_3	2217.0	30.0	99.4	2228.8	30.1	100.7
	WRF_fx5	1879.4	25.4	84.3	1418.5	19.2	64.1
	WRF_fx4	1489.8	20.2	66.8	926.0	12.5	41.9
	WRF_fx3	977.2	13.2	43.8	551.6	7.5	24.9
	WRF_5fx5	1879.6	25.4	84.3	1418.8	19.2	64.1
	WRF_5fx4	1489.9	20.2	66.8	926.1	12.5	41.9
	WRF_5fx3	977.3	13.2	43.8	551.4	7.5	24.9
	WRF_4fx5	1876.1	25.4	84.2	1414.8	19.2	64.1
	WRF_4fx4	1489.5	20.1	66.8	925.8	12.5	41.8
	WRF_4fx3	972.1	13.1	43.6	545.8	7.4	24.7
	WRF_3fx5	1872.2	25.3	84.0	1433.2	19.4	64.8
	WRF_3fx4	1486.5	20.1	66.7	937.6	12.7	42.4
	WRF_3fx3	978.2	13.2	43.9	560.5	7.6	25.3

* Notes: Relative compression ratio = (compressed size of precision-reduced configurations */* compressed size of WRF_bl).

**Table 6.** Compression performance of a 5 d WRF input file for the full-precision data and data retaining 5, 4, and 3 significant digits, respectively. Numbers in parentheses indicate specific configurations: (compression level, number of threads) for Zstd, and (deflation level) for zlib.

	Compressor	Full Precision	5 digits	4 digits	3 digits
Size (% of uncompressed)	bzip2	75.11	52.98	33.11	18.56
	gzip	75.61	69.33	55.04	34.95
	zlib (1)	75.08	68.18	55.78	38.59
	zlib (9)	74.8	69.1	54.42	33.63
	Zstd (3,1)	76.87	73.19	58.08	36.7
	Zstd (7,1)	76.1	65.29	50.95	31.16
	Zstd (19,1)	74.16	58.32	43.15	25.54
	Zstd (19,8)	74.14	58.27	43.08	25.47
Compression (s)	bzip2	821.83	713.64	635.17	656.6
	gzip	811.32	609.17	760.91	893.29
	zlib (1)	301	247	199	143
	zlib (9)	571	516	743	1289
	Zstd (3,1)	34.48	64.47	67.52	64.35
	Zstd (7,1)	169.25	293.75	266.06	189.56
	Zstd (19,1)	3544.12	2905.94	3301.46	3345.15
	Zstd (19,8)	401.65	502.2	536.14	521.97
Decompression (s)	bzip2	468.92	404.08	308.98	239.09
	gzip	76.91	77.76	76.8	63.09
	zlib (1)	0	0	0	0
	zlib (9)	0	0	0	0
	Zstd (3,1)	10.96	13.39	14.56	13.3
	Zstd (7,1)	12.17	15.76	15.21	12.82
	Zstd (19,1)	16.11	23.98	20.14	13.09
	Zstd (19,8)	16.71	23.89	20.43	13.23

**Table 7.** Compression performance of a 1 d WRF output file for the full-precision data and data retaining 5, 4, and 3 significant digits, respectively. Numbers in parentheses indicate specific configurations: (compression level, number of threads) for Zstd, and (deflation level) for zlib.

	Compressor	Full Precision	5 digits	4 digits	3 digits
Size (% of uncompressed)	bzip2	30.46	19.68	12.88	7.65
	gzip	30.66	25.93	20.66	13.58
	zlib (1)	30.98	26.13	21.3	15.08
	zlib (9)	30.34	25.63	20.18	12.96
	Zstd (3,1)	30.41	26.6	21.26	14.34
	Zstd (7,1)	30.18	24.27	18.95	12.42
	Zstd (19,1)	29.79	21.82	16.12	10.17
	Zstd (19,8)	29.78	21.81	16.11	10.16
Compression (s)	bzip2	940.15	855.28	839.07	879.25
	gzip	410.83	569.1	578.58	576.38
	zlib (1)	253	207	176	142
	zlib (9)	614	749	905	1125
	Zstd (3,1)	38.9	54.08	62.68	45.06
	Zstd (7,1)	116.27	186.48	188.44	130.43
	Zstd (19,1)	1664.3	1711.5	1963.4	2517.14
	Zstd (19,8)	326.24	337.98	352.54	325.12
Decompression (s)	bzip2	361.47	306.41	251.31	206.12
	gzip	99.43	101.65	99.04	90.94
	zlib (1)	0	0	0	0
	zlib (9)	0	0	0	0
	Zstd (3,1)	10.99	12.19	12.63	11.79
	Zstd (7,1)	11.18	13.51	12.69	11.24
	Zstd (19,1)	12.67	17.66	15.44	11.28
	Zstd (19,8)	12.66	17.6	15.03	11.34

**Table 8.** Statistical summaries of grid-scale AD and domain-scale SSIM relative to WRF_bl.

Variable	Precision configuration	Mean AD	Maximum AD	Mean SSIM	Minimum SSIM	1st Percentile SSIM
*T*_2_ (K)	WRF_5	0.0581798	14.25198	0.9939092	0.9630990	0.9709463
	WRF_4	0.0594887	21.44626	0.9938635	0.9626499	0.9705832
	WRF_3	0.0894812	21.11664	0.9921307	0.9605753	0.9682225
	WRF_fx5	0.0025001	0.0050000	0.9999945	0.9995806	0.9998766
	WRF_fx4	0.0250000	0.0500000	0.9997513	0.9981659	0.9991125
	WRF_fx3	0.2500606	0.5000000	0.9713302	0.9423122	0.9506720
*Q*_2_ (kg kg^−1^)	WRF_5	0.0000477	0.0134958	0.9898861	0.9457591	0.9574129
	WRF_4	0.0000486	0.0125344	0.9897875	0.9471009	0.9578402
	WRF_3	0.0000677	0.0120278	0.9853344	0.9410679	0.9523994
	WRF_fx5	0.0000001	0.0000005	0.9999999	0.9999989	0.9999996
	WRF_fx4	0.0000010	0.0000050	0.9999960	0.9999886	0.9999907
	WRF_fx3	0.0000105	0.0000500	0.9994877	0.9988874	0.9989862
WS10 (m s^−1^)	WRF_5	0.0717549	22.79240	0.9779398	0.8997450	0.9211134
	WRF_4	0.0726728	21.88551	0.9777849	0.8985054	0.9208920
	WRF_3	0.1003181	18.21674	0.9678928	0.8853601	0.9100209
	WRF_fx5	0.0000324	0.0007067	0.9999999	0.9999995	0.9999998
	WRF_fx4	0.0003241	0.0070658	0.9999995	0.9999979	0.9999986
	WRF_fx3	0.0032415	0.0705795	0.9999494	0.9998048	0.9998650
PSFC (Pa)	WRF_5	1.6698395	811.9844	0.9999944	0.9999550	0.9999727
	WRF_4	1.6841504	878.2109	0.9999943	0.9999399	0.9999720
	WRF_3	2.3376082	804.9219	0.9999937	0.9999480	0.9999702
	WRF_fx5	1.2597312	5.0000	0.9999974	0.9998826	0.9999572
	WRF_fx4	12.595335	50.0000	0.9997887	0.9991961	0.9994622
	WRF_fx3	127.72333	500.0000	0.9947414	0.9909650	0.9919420
Precipitation (mm h^−1^)	WRF_5	0.0443069	130.6014	0.9816086	0.8783776	0.9302602
	WRF_4	0.0444704	139.8082	0.9815308	0.8769866	0.9299442
	WRF_3	0.0495302	145.4452	0.9782254	0.8696560	0.9271690
	WRF_fx5	0.0014635	0.195557	0.9998999	0.9946446	0.9983964
	WRF_fx4	0.0105326	1.972900	0.9963600	0.9353753	0.9608235
	WRF_fx3	0.5544670	19.90356	0.9632875	0.7775496	0.8290028

**Table 9.** Categorical verification metrics for hourly precipitation across different precision-reduced configurations relative to the WRF_bl.

Configuration	ZPR (%)	POD (%)	FAR (%)	CSI	Bias
WRF_5	98.7086	92.1787	7.8366	0.8548	1.00017
WRF_4	98.7078	92.1526	7.8431	0.8545	0.99995
WRF_3	98.5641	91.2276	8.7198	0.8391	0.99942
WRF_fx5	99.8289	99.2410	1.0357	0.9822	1.00280
WRF_fx4	98.4663	94.3254	8.9820	0.8629	1.03634
WRF_fx3	99.1221	71.9411	6.8957	0.6830	0.77269
WRF_5fx5	98.6073	91.8428	8.4280	0.8468	1.00296
WRF_5fx4	97.4814	88.3594	14.7486	0.7664	1.03646
WRF_5fx3	98.5405	68.4151	11.4639	0.6285	0.77274
WRF_4fx5	98.6069	91.8190	8.4321	0.8466	1.00274
WRF_4fx4	97.4812	88.3404	14.7522	0.7663	1.03628
WRF_4fx3	98.5400	68.4076	11.4680	0.6284	0.77269
WRF_3fx5	98.4697	90.9292	9.2680	0.8320	1.00217
WRF_3fx4	97.3682	87.6111	15.4209	0.7554	1.03585
WRF_3fx3	98.4717	67.9713	12.0081	0.6220	0.77247

* Note: A threshold of 0.1 mm h^−1^ is applied to distinguish between dry and wet grids. ZPR is the Zero Preservation Ratio, POD denotes the Probability of Detection, FAR is the False Alarm Ratio, CSI is the Critical Success Index, and Bias represents the Frequency Bias.

## Data Availability

A tool for formatting data to a user-specified number of significant digits is available for download on Zenodo at https://doi.org/10.5281/zenodo.19199806 (Wong and Wu, 2026). The download includes the full source code, a run script, and detailed usage instructions. Surface meteorological data (wind speed, temperature, and humidity) were sourced from the NOAA National Centers for Environmental Information (NCEI) Global Hourly dataset, accessible at: https://www.ncei.noaa.gov/data/global-hourly/archive/csv/ (last access: 28 September 2025). Precipitation data were obtained from the GPM IMERG Final Precipitation L3 product (Version 07), with daily and hourly data available via https://doi.org/10.5067/GPM/IMERGDF/DAY/07 (Huffman et al., 2023b) and https://doi.org/10.5067/GPM/IMERG/3B-HH/07 (Huffman et al., 2023a), respectively. We acknowledge the use of imagery from NASA Worldview, operated by NASA’s Earth Observing System Data and Information System (EOSDIS). The maps were obtained from https://worldview.earthdata.nasa.gov/ (last access: 28 September 2025).

## Data availability.

The processed analytic datasets that support the main findings of this study are openly available on Zenodo at https://doi.org/10.5281/zenodo.19781791 (Wu and Wong, 2026).
