# Supplementary material for: Technical note: A flexible framework for precision reduction of WRF inputs and outputs to balance storage efficiency and scientific fidelity
Source: Atmos Chem Phys. Author manuscript; Available in PMC 2026 Jul 1. (PMC13316787; doi:10.5194/acp-26-7261-2026)
Supplement: Supplement1 [file NIHMS2190268-supplement-Supplement1.pdf]

Supplement of Atmos. Chem. Phys., 26, 7261–7285, 2026
https://doi.org/10.5194/acp-26-7261-2026-supplement
© Author(s) 2026. CC BY 4.0 License.

Atmospheric Chemistry and Physics
Open Access

Supplement of

Technical note: A flexible framework for precision reduction of WRF inputs and outputs to balance storage efficiency and scientific fidelity

Shang Wu et al.

Correspondence to: David C. Wong (wong.david-c@epa.gov) and Jiandong Wang (jiandong.wang@nuist.edu.cn)

The copyright of individual parts of the supplement might differ from the article licence.

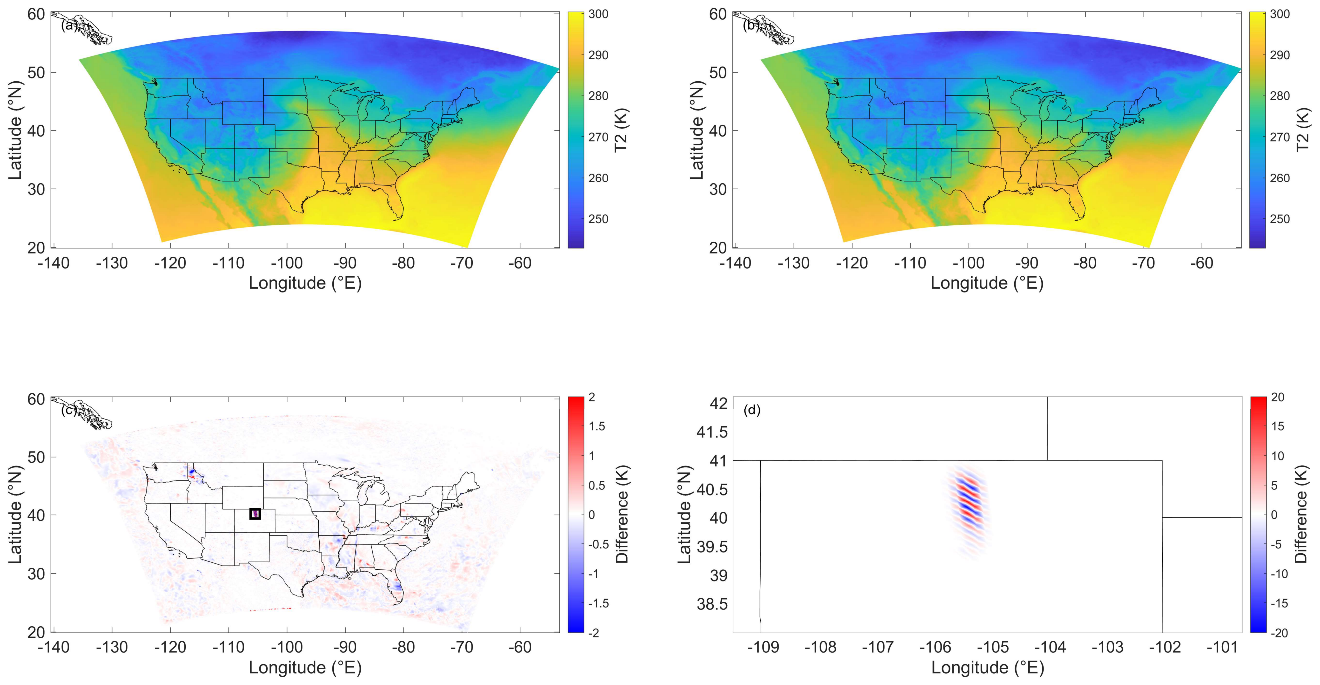

Figure S1: Spatial visualization of T2 at the time step exhibiting the maximum AD. (a) WRF_bl, (b) WRF_3, (c) the spatial difference fields (WRF_3 minus WRF_bl), and (d) a locally zoomed-in view of the difference field corresponding to the bold black box in (c). The black square marks the precise location of the maximum AD (21.12 K). At this specific time step, the domain-wide SSIM is 0.998.

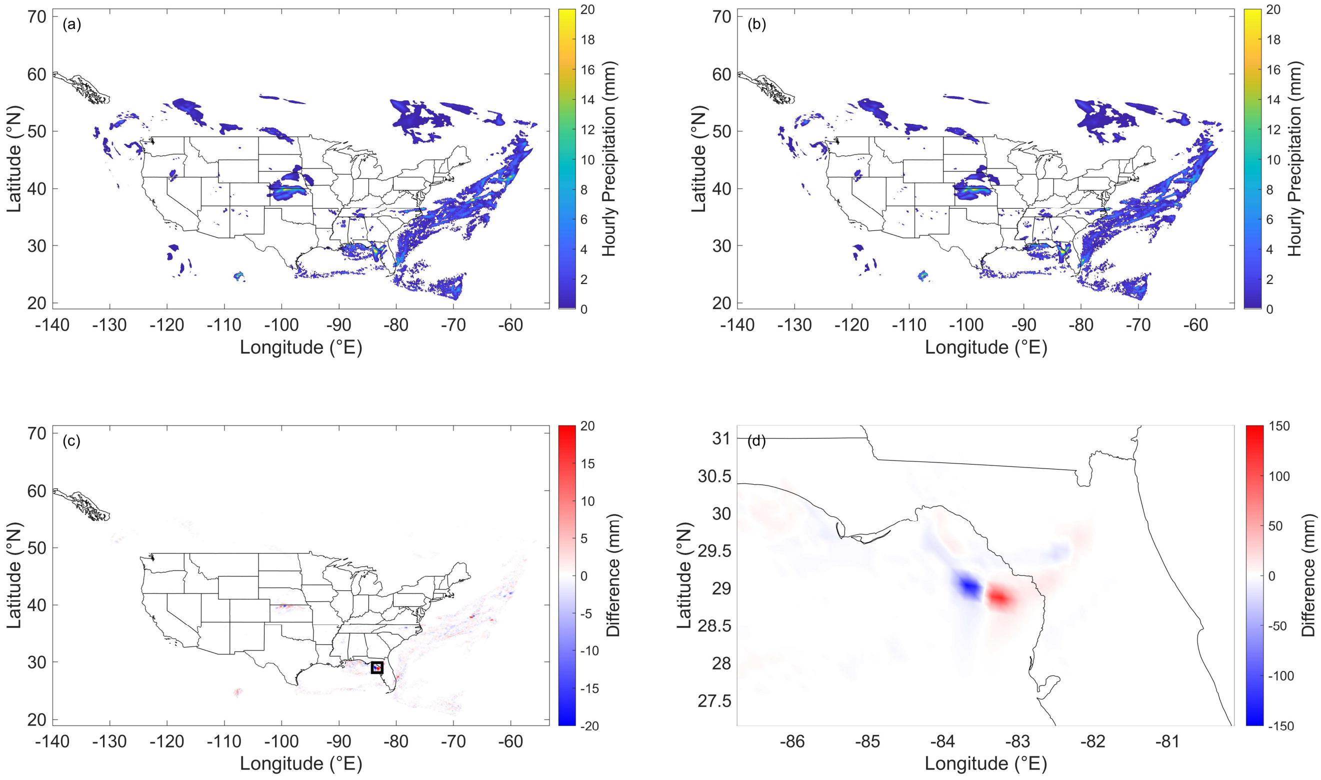

Figure S2: Spatial visualization of hourly precipitation at the time step exhibiting the maximum AD. (a) WRF_bl, (b) WRF_3, (c) the spatial difference fields (WRF_3 minus WRF_bl), and (d) a locally zoomed-in view of the difference field corresponding to the bold black box in (c). The black square in panel (c) pinpoints the exact location of the maximum AD (145.45 mm). At this specific time step, the domain-wide SSIM is 0.986.

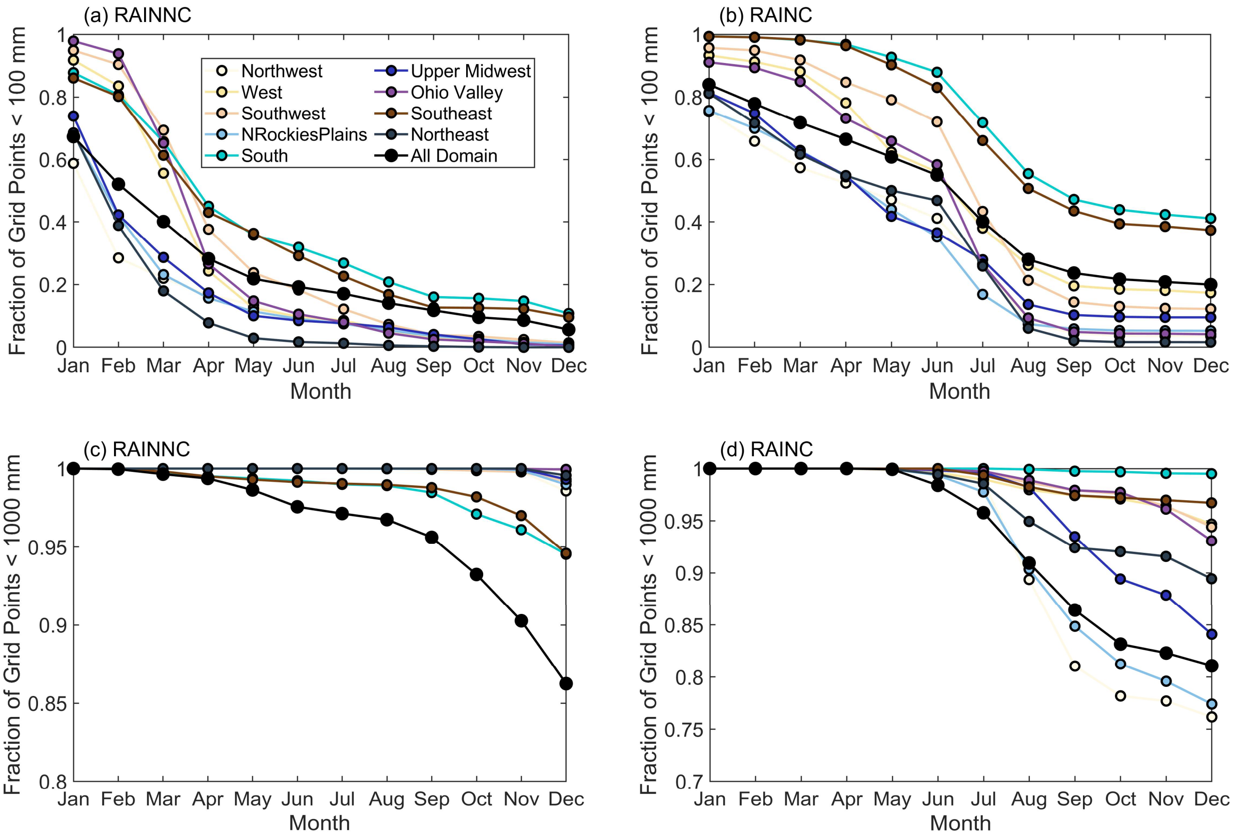

Figure S3: Monthly evolution of the fraction of grid points with accumulated non-convective (RAINNC; a, c) and convective (RAINC; b, d) precipitation remaining below the 100 mm (top row) and 1000 mm (bottom row) thresholds. The lines denote different climate regions and the entire WRF simulation domain average.

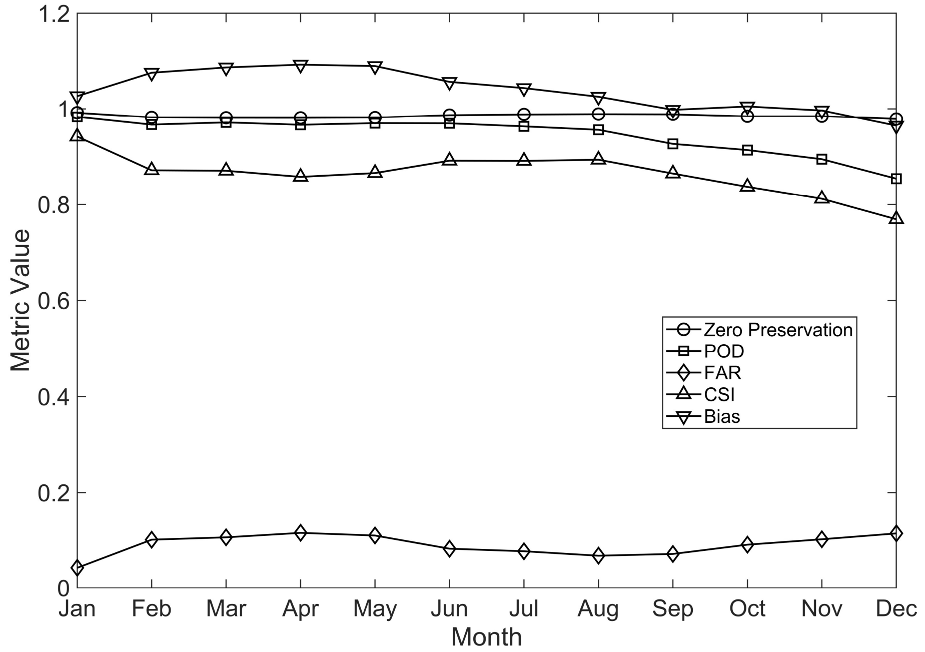

Figure S4: temporal evolution of precipitation occurrence metrics under the WRF_fx4 configuration.

Table S1. Supplementary statistical summaries of grid-scale AD and domain-scale SSIM relative to WRF_bl.

Variable	Precision Configuration	Mean AD	Maximum AD	Mean SSIM	Minimum SSIM	1th Percentile SSIM
T2 (K)	WRF_5fx5	0.0585092	14.24808	0.9941464	0.9638181	0.9714917
	WRF_4fx5	0.0596670	21.44217	0.9941094	0.9631336	0.9711497
	WRF_3fx5	0.0895583	21.11215	0.9923728	0.9609654	0.9688216
	WRF_5fx4	0.0698946	14.28809	0.9935838	0.9624803	0.9705521
	WRF_4fx4	0.0704955	21.43216	0.9935364	0.9620183	0.9701092
	WRF_3fx4	0.0954634	21.13217	0.9917775	0.9599726	0.9677429
	WRF_5fx3	0.2695719	13.88809	0.9653397	0.9223217	0.9361422
	WRF_4fx3	0.2697850	21.03217	0.9652849	0.9204355	0.9355961
	WRF_3fx3	0.2765166	21.03217	0.9634103	0.9183630	0.9325463
Q2 (kg kg^{-1})	WRF_5fx5	0.0000477	0.0134958	0.9898865	0.9457601	0.9574135
	WRF_4fx5	0.0000485	0.0125344	0.9897912	0.9471013	0.9578403
	WRF_3fx5	0.0000677	0.0120282	0.9853347	0.9410694	0.9523994
	WRF_5fx4	0.0000478	0.0134958	0.9898820	0.9457523	0.9574100
	WRF_4fx4	0.0000486	0.0125345	0.9897867	0.9470969	0.9578386
	WRF_3fx4	0.0000678	0.0120302	0.9853302	0.9410619	0.9523916
	WRF_5fx3	0.0000509	0.0134955	0.9893829	0.9450131	0.9566665
	WRF_4fx3	0.0000515	0.0125355	0.9892875	0.9462982	0.9571850
	WRF_3fx3	0.0000692	0.0120402	0.9848379	0.9403619	0.9516387
WS10 (m s^{-1})	WRF_5fx5	0.0717551	22.79204	0.9779398	0.8997450	0.9211135
	WRF_4fx5	0.0726427	21.88501	0.9777967	0.8985056	0.9208920
	WRF_3fx5	0.1003181	18.21677	0.9678928	0.8853604	0.9100208
	WRF_5fx4	0.0717656	22.79304	0.9779393	0.8997451	0.9211132
	WRF_4fx4	0.0726502	21.88602	0.9777962	0.8985054	0.9208917
	WRF_3fx4	0.1003208	18.21657	0.9678922	0.8853602	0.9100215
	WRF_5fx3	0.0723285	22.77304	0.9778875	0.8997257	0.9210765
	WRF_4fx3	0.0731597	21.83602	0.9777444	0.8984851	0.9208500
	WRF_3fx3	0.1005638	18.21553	0.9678424	0.8853513	0.9099925
PSFC (Pa)	WRF_5fx5	2.3953139	811.6875	0.9999967	0.9998835	0.9999513
	WRF_4fx5	2.4031211	877.7578	0.9999966	0.9998866	0.9999513
	WRF_3fx5	2.8537383	804.6641	0.9999966	0.9998521	0.9999504
	WRF_5fx4	13.063542	807.6875	0.9997818	0.9991977	0.9994573
	WRF_4fx4	13.068286	873.7578	0.9997816	0.9991989	0.9994567
	WRF_3fx4	13.184773	800.6641	0.9997793	0.9992093	0.9994533
	WRF_5fx3	127.81349	930.6406	0.9947367	0.9909427	0.9919432
	WRF_4fx3	127.81529	938.3750	0.9947364	0.9909418	0.9919378
	WRF_3fx3	127.82901	886.9766	0.9947321	0.9909020	0.9919389
Precipitation (mm h^{-1})	WRF_5fx5	0.0448970	130.5917	0.9815108	0.8783125	0.9301871
	WRF_4fx5	0.0450515	139.8071	0.9814382	0.8768666	0.9299014
	WRF_3fx5	0.0500592	145.4512	0.9781288	0.8695480	0.9271399
	WRF_5fx4	0.0501815	131.0017	0.9781844	0.8748295	0.9271685
	WRF_4fx4	0.0503287	139.807	0.9781112	0.8739549	0.9264433

WRF_3fx4	0.0550365	145.4011	0.9748645	0.8666406	0.9233193
WRF_5fx3	0.0829424	135.6245	0.9481691	0.7706791	0.8223788
WRF_4fx3	0.0830692	140.207	0.9481080	0.7686988	0.8232154
WRF_3fx3	0.0865763	144.8011	0.9455325	0.7724452	0.8195075

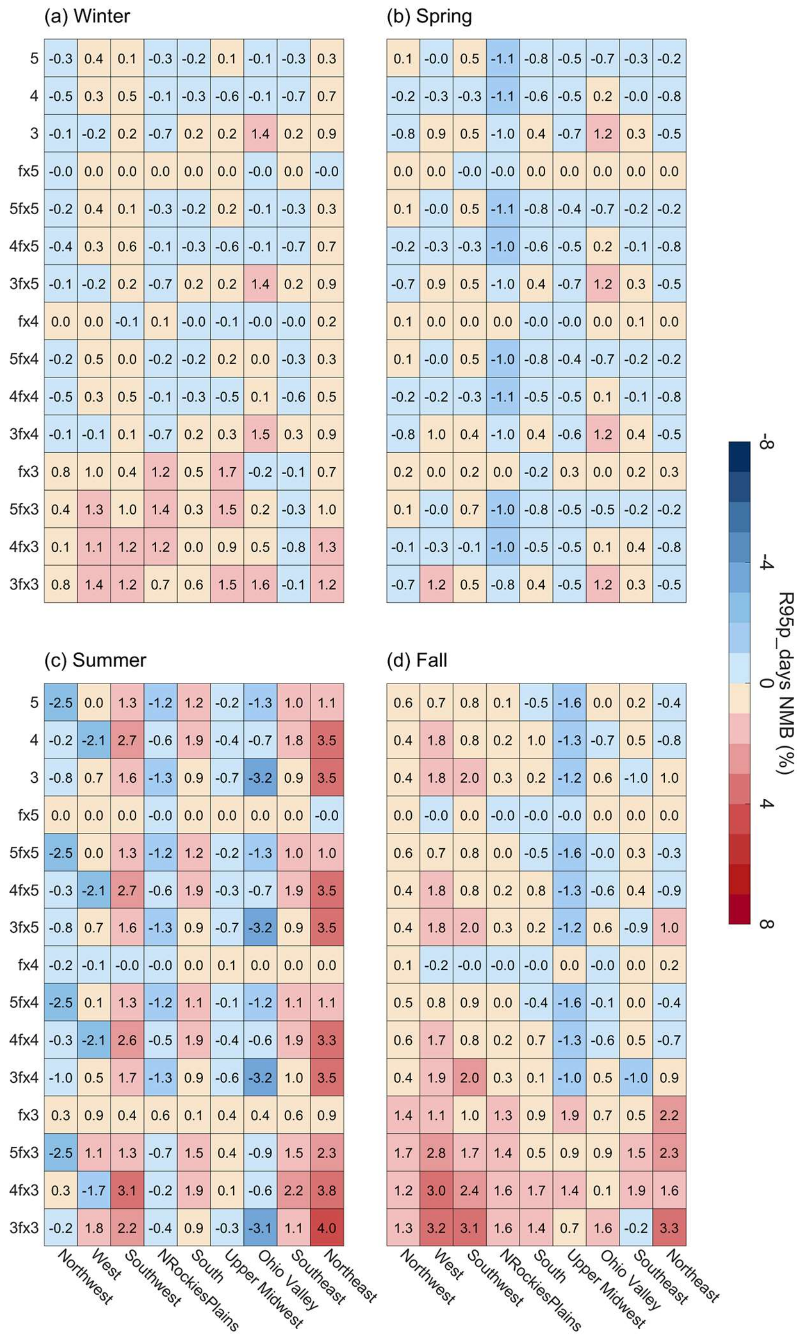

Figure S5: Seasonal and regional NMB of R95p_days (a–d).

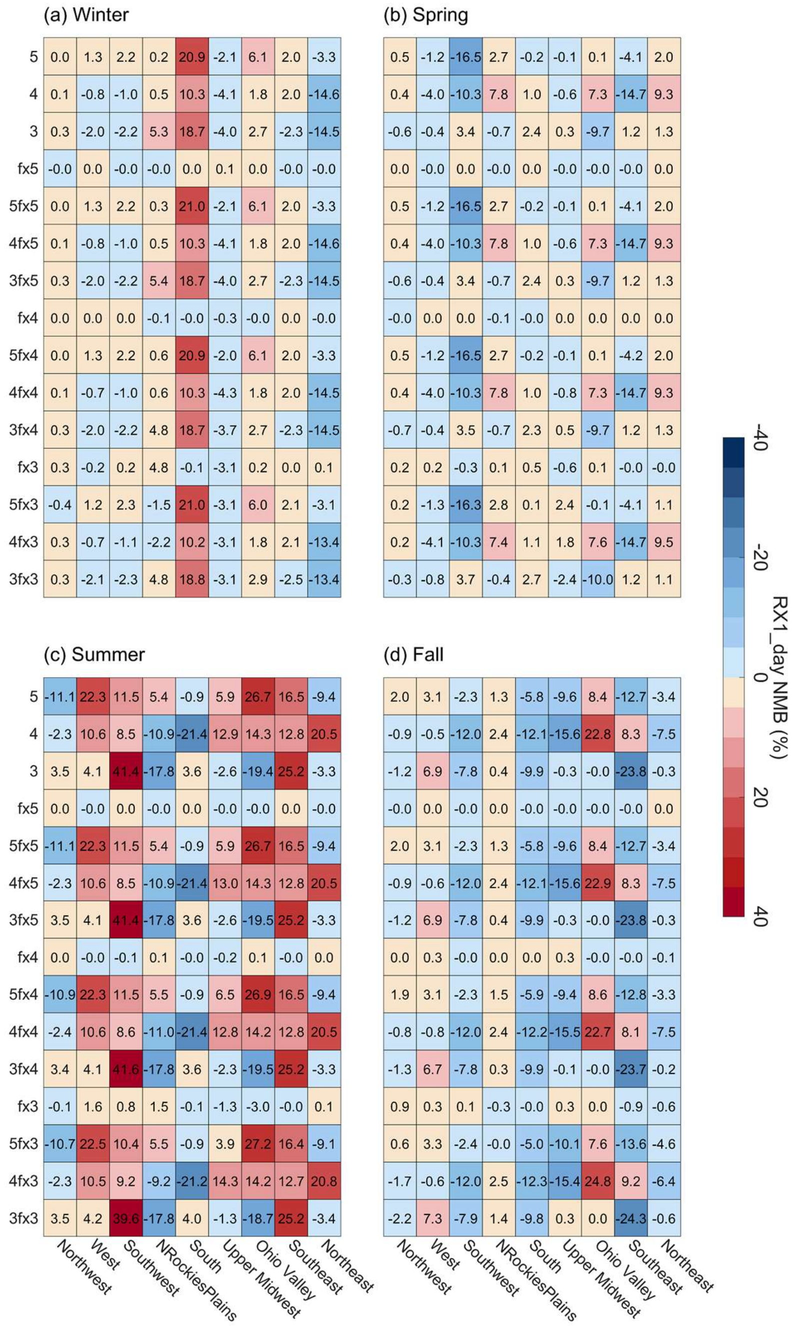

Figure S6: Seasonal and regional NMB of RX1_day (a–d).

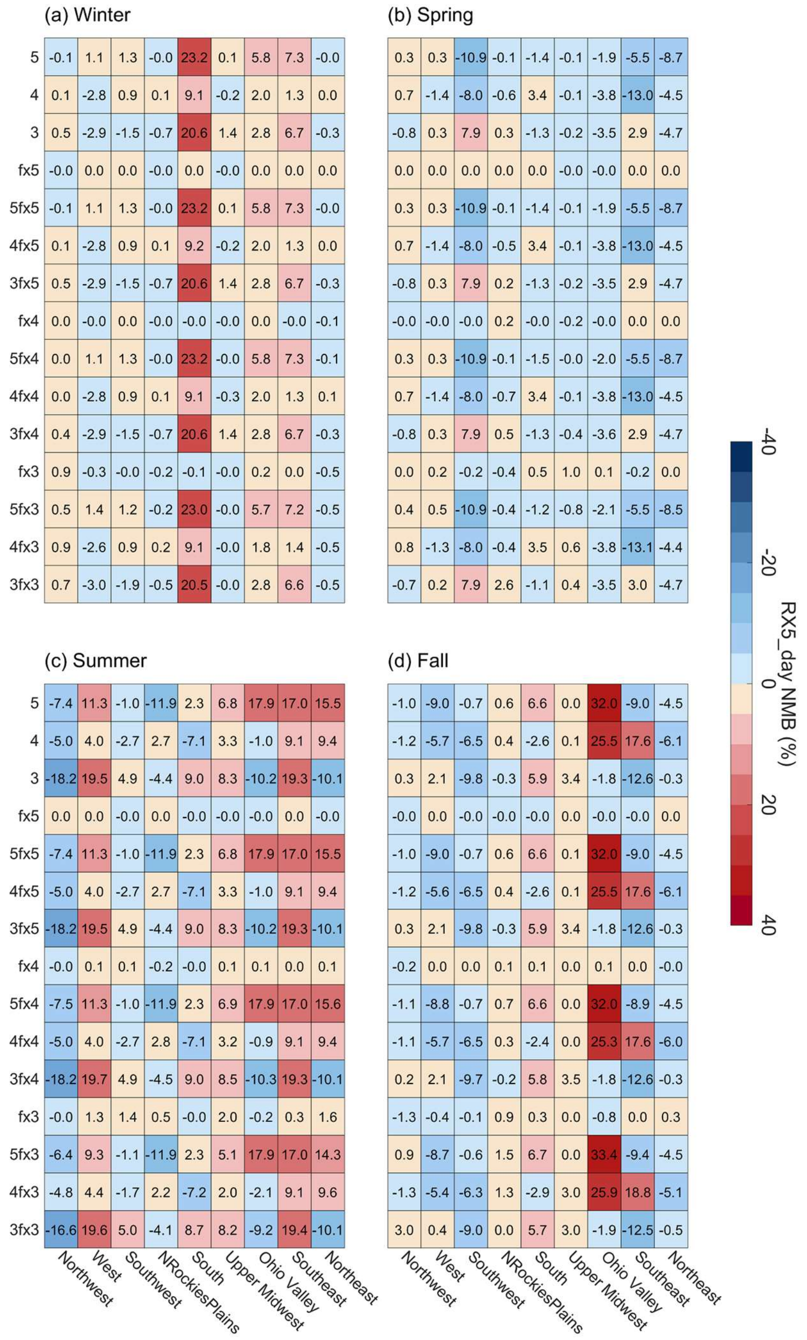

Figure S7: Seasonal and regional NMB of RX5_day (a–d).

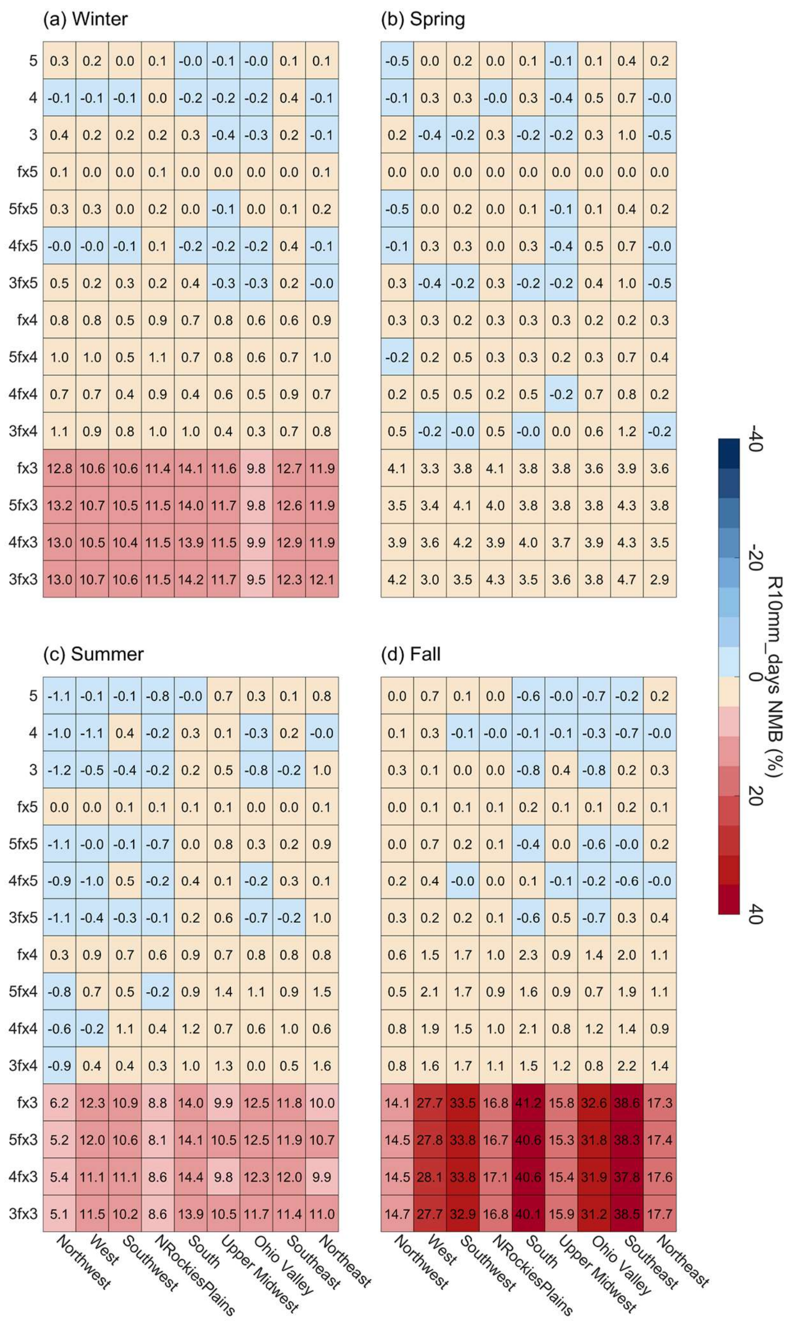

Figure S8: Seasonal and regional NMB of R10mm_days (a–d).

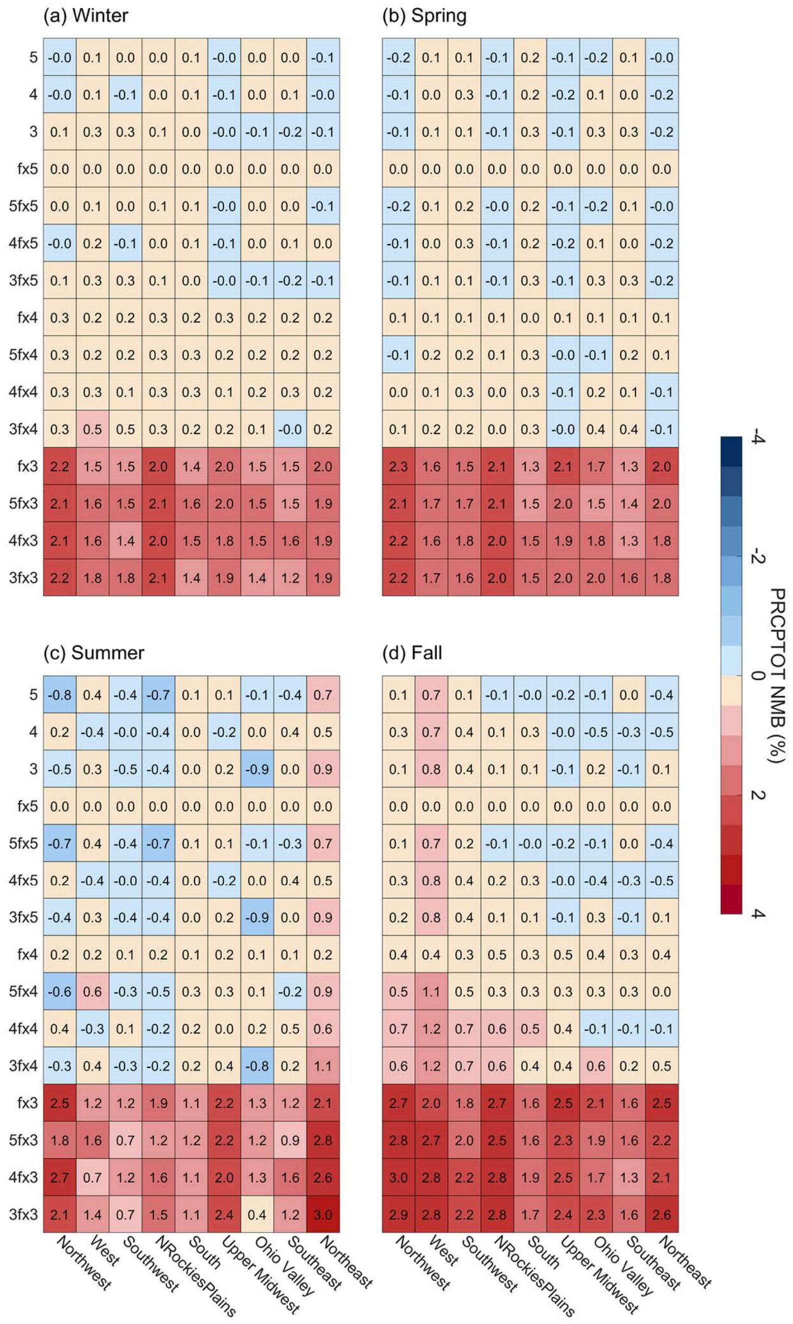

Figure S9: Seasonal and regional NMB of PRCPTOT (a–d).

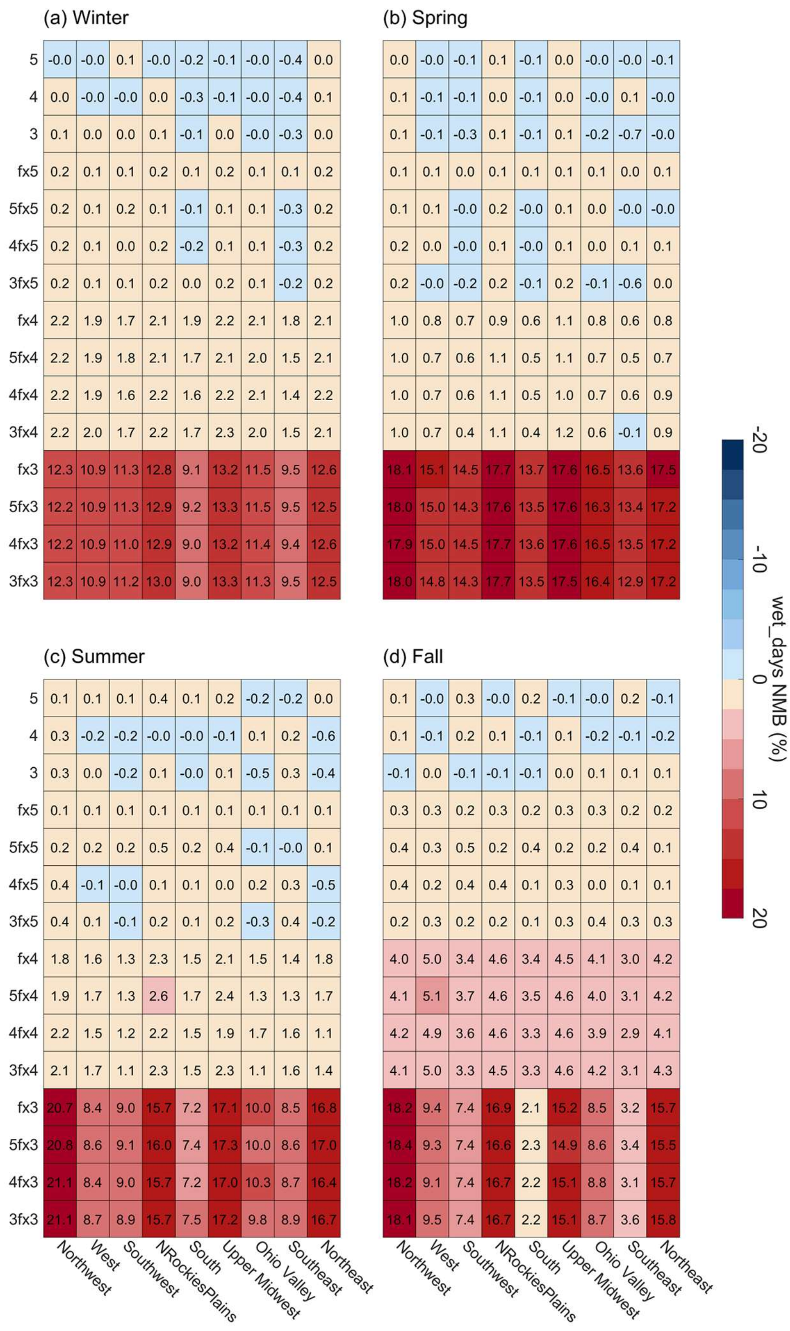

Figure S10: Seasonal and regional NMB of wet_days (a–d).
